# RNAi of the sesquiterpene cyclase gene for phytoalexin production impairs pre‐ and post‐invasive resistance to potato blight pathogens

**DOI:** 10.1111/mpp.12802

**Published:** 2019-04-16

**Authors:** Miki Yoshioka, Ayako Adachi, Yutaka Sato, Noriyuki Doke, Tatsuhiko Kondo, Hirofumi Yoshioka

**Affiliations:** ^1^ Graduate School of Bioagricultural Sciences Nagoya University Chikusa Nagoya 464‐8601 Japan; ^2^ National Institute of Genetics Yata 1111, Mishima Shizuoka 411‐8540 Japan

**Keywords:** Phytoalexin, plant immunity, potato blight pathogen, RNA interference, sesquiterpene cyclase

## Abstract

Potato antimicrobial sesquiterpenoid phytoalexins lubimin and rishitin have been implicated in resistance to the late blight pathogen, *Phytophthora infestans* and early blight pathogen, *Alternaria solani*. We generated transgenic potato plants in which sesquiterpene cyclase, a key enzyme for production of lubimin and rishitin, is compromised by RNAi to investigate the role of phytoalexins in potato defence. The transgenic tubers were deficient in phytoalexins and exhibited reduced post‐invasive resistance to an avirulent isolate of *P. infestans*, resulting in successful infection of the first attacked cells without induction of cell death. However, cell death was observed in the subsequently penetrated cells. Although we failed to detect phytoalexins and antifungal activity in the extract from wild‐type leaves, post‐invasive resistance to avirulent *P. infestans* was reduced in transgenic leaves. On the other hand, *A. solani* frequently penetrated epidermal cells of transgenic leaves and caused severe disease symptoms presumably from a deficiency in unidentified antifungal compounds. The contribution of antimicrobial components to resistance to penetration and later colonization may vary depending on the pathogen species, suggesting that sesquiterpene cyclase‐mediated compounds participate in pre‐invasive resistance to necrotrophic pathogen *A. solani* and post‐invasive resistance to hemibiotrophic pathogen *P. infestans*.

## Introduction

Plants sense the presence of potential pathogens by detecting pathogen‐associated molecular patterns (PAMPs) via pattern recognition receptors (PRRs), then initiate a first layer of defence responses by pattern‐triggered immunity (PTI), which blocks the vast majority of plant pathogens (Jones and Dangl, 2006; Macho and Zipfel, 2014). In turn, pathogens evolved effector molecules to overcome the PTI and infect host plants. The second layer of immunity, effector‐triggered immunity (ETI), results from the recognition of pathogen effector molecules by host resistance proteins, which are often nucleotide‐binding leucine‐rich repeat (NB‐LRR or NLR) proteins. *NLR* gene‐mediated resistance triggers a strong gene‐for‐gene resistance that induces generation of reactive nitrogen and oxygen species, hypersensitive response (HR) cell death and accumulation of antimicrobial phytoalexins (Doke *et al*., 1996).

Late blight, caused by the notorious oomycete *Phytophthora infestans* is a highly devastating disease of potato (*Solanum tuberosum*) and tomato (*Solanum lycopersicum*). Inoculation of potato tubers with an avirulent isolate of *P. infestans* triggers accumulation of sesquiterpenoid phytoalexins, such as lubimin and rishitin, in tubers. Although potato leaves are the primary infection sites under natural conditions, sesquiterpenoid phytoalexins do not accumulate in detectable amounts in leaves (Rohwer *et al*., 1987).

**Figure 1:**
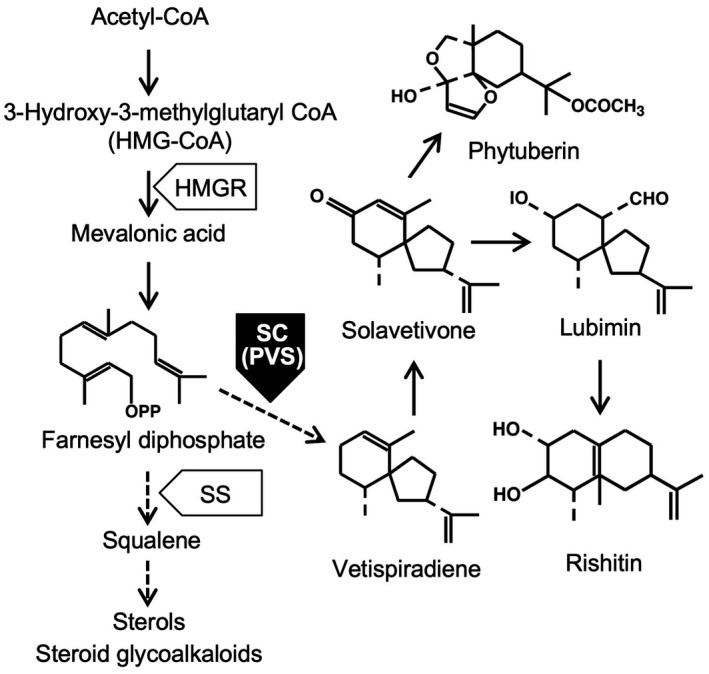
Scheme of sesquiterpenoid phytoalexin synthesis in potato (*Solanum tuberosum*). Potato vetispiradiene synthase (PVS), which is sesquiterpene cyclase, is a key branch enzyme of the isoprenoid pathway for the synthesis of sesquiterpenoid phytoalexins, solavetivone, phytuberin, lubimin and rishitin. Lubimin and rishitin mainly accumulate in tubers. HMGR; HMG‐CoA reductase, SS; squalene synthetase, SC; sesquiterpene cyclase.

Sesquiterpenoid phytoalexins are synthesized via the mevalonate pathway. HMG‐CoA is converted to mevalonate by 3‐hydroxy‐3‐methylglutaryl CoA reductase (HMGR) as the first step of the synthesis of isoprenoids (Fig. 1). Sesquiterpene cyclase (SC) is a key branch enzyme of the isoprenoid pathway for the production of sesquiterpenoid phytoalexins (Back and Chappell, 1995; Zook and Kuć, 1991). Cyclization of farnesyl diphosphate to vetispiradiene catalyzed by potato vetispiradiene synthase (PVS), which is an SC, produces antimicrobial solavetivone, a precursor of phytuberin, lubimin and rishitin (Stoessl *et al*., 1976). Wound‐induced expression of *HMGR1* and *squalene synthase* genes, which participate in sterol and steroid glycoalkaloid biosynthesis, are suppressed in favour of sesquiterpenoid phytoalexin synthesis during immune responses (Choi *et al*., 1992; Yoshioka *et al*., 1999, 2001). *PVS* is encoded by a multiple‐gene family (*PVS1* to *PVS4*). Infection of *P. infestans* causes transient increases in transcript levels of *PVS* in potato tubers during not only incompatible, but also compatible interactions (Yoshioka *et al*., 1999). Amongst the *PVS* genes, only *PVS3* was markedly induced in leaves during both interactions (Yamamizo *et al*., 2006). The *PVS3* gene comprises seven exons, similar to other solanaceous *SC* genes, *Nicotiana tabacum* and pepper (*Capsicum annuum*) *5‐epi*‐*aristolochene synthase* (*EAS*), which encode a key enzyme for capsidiol synthesis in their leaves, whereas *PVS1*, *PVS2* and *PVS4* contain only six exons (Fig. S1; Yamamizo *et al*., 2006). The phosphorylated WRKY8 transcription factor in *Nicotiana benthamiana* positively regulates expression of *HMGR2* downstream of defence‐related mitogen‐activated protein kinases (MAPK), salicylic acid‐induced protein kinase (SIPK) and wound‐induced protein kinase (WIPK) after infection by *P. infestans* (Ishihama *et al*., 2011). Because the *PVS3* promoter is also activated by the same MAPK cascades (Yamamizo *et al*., 2006), sesquiterpenoid phytoalexin biosynthesis possibly could be regulated by MAPK cascades similar to camalexin, an indole alkaloid phytoalexin of *Arabidopsis thaliana* (Ren *et al*., 2008).

In general, phytoalexins are known to play important roles in post‐invasive resistance by inhibiting pathogen growth after penetration into the attacked cell. In some pathosystems, pre‐existing antimicrobial compounds contribute to the pre‐invasive resistance by inhibiting penetration from appressoria. *Phytoalexin deficient 3* (*PAD3*) encodes CYP71B15 P450 monooxygenase and catalyzes the final step of camalexin biosynthesis in *Arabidopsis* *thaliana* (Schuhegger *et al*., 2006; Zhou *et al*., 1999). The Arabidopsis *pad3* mutant is more susceptible than wild‐type plants to the necrotrophic pathogen *Alternaria brassicicola* (Thomma *et al*., 1999). A non‐adapted hemibiotrophic pathogen *Colletotrichum gloeosporioides* penetrates cells of the Arabidopsis *pen2* mutant, which lacks pre‐invasive resistance, without invasive hyphal colonization. However, the *pen2 pad3* double mutant is susceptible to *C. gloeosporioides*, indicating that camalexin is involved in post‐invasive resistance to the pathogen **(**Hiruma *et al*., 2013). In addition, full‐size ABCG transporters are involved in the export of constitutively produced diterpenes for pre‐invasive defence and newly synthesized capsidiol for post‐invasive defence in *N. benthamina* against *P. infestans* (Shibata *et al*., 2016).

Here, we investigated the role of PVS‐mediated compounds in defence against the near‐obligate hemibiotrophic *P. infestans* (Erwin and Ribeiro, 1996; Fry, 2008) and the necrotrophic potato early blight pathogen, *Alternaria solani*, which also causes a devastating disease on leaves of potato and tomato. Transgenic potato leaves with *PVS*‐silenced by RNA interference (RNAi) had greater susceptibility to both *P. infestans* and *A. solani*. We showed that lubimin and rishitin in tubers participate in ETI‐triggered hypersensitive response (HR) in response to *P. infestans*. Molecular analyses using *PVS*‐silenced potato plants suggest that PVS has a role in producing antimicrobial components in potato leaves and that PVS‐mediated compounds are involved in pre‐invasive resistance to *A. solani* and post‐invasive resistance to *P. infestans*.

## Results

### Phytoalexins did not accumulate in *PVS*‐silenced transgenic potato tubers after inoculation with *P. infestans*

**Figure 2:**
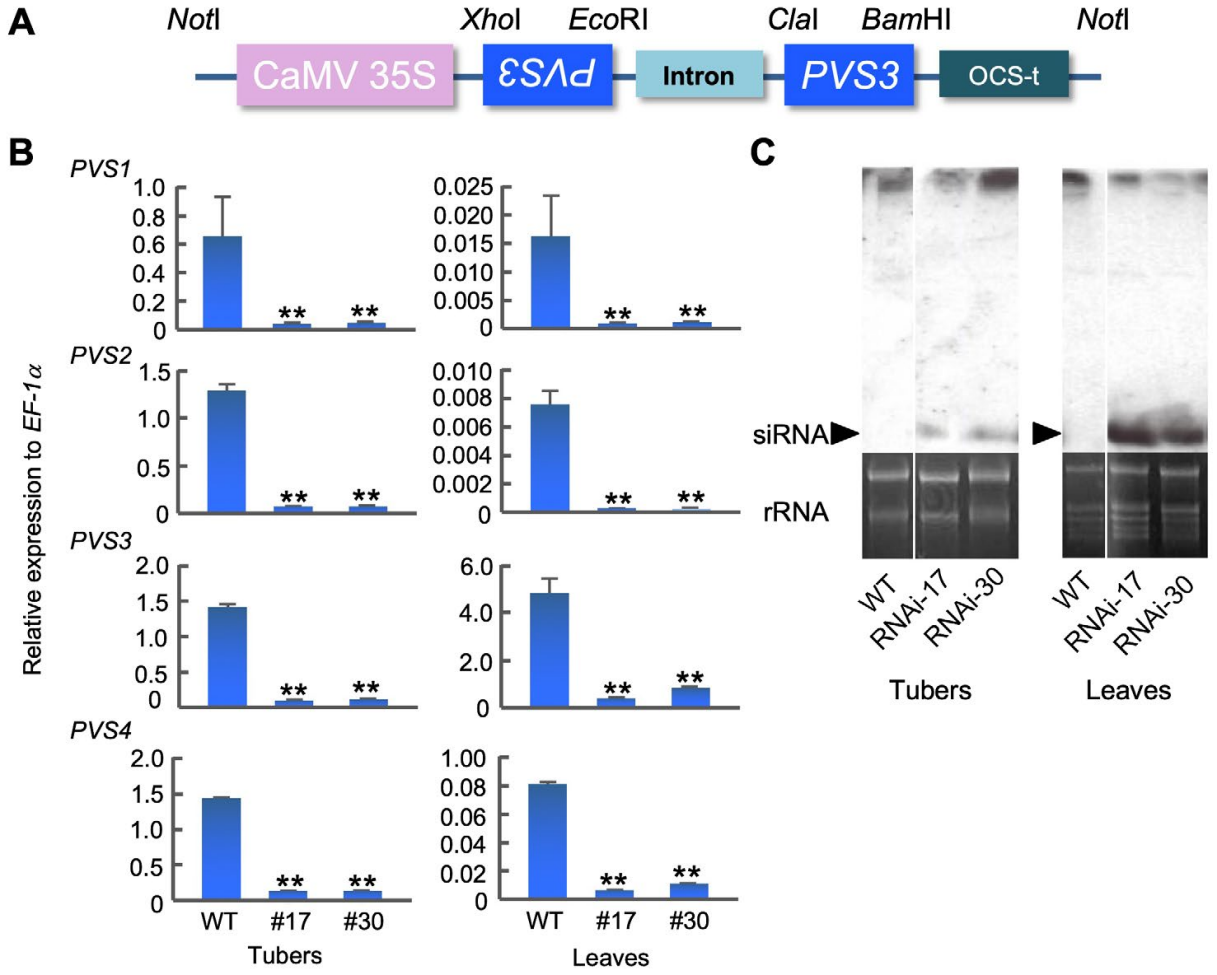
RNAi silencing of *PVS* genes in transgenic potato (*Solanum tuberosum*) plants expressing dsRNA of *PVS3*. (A) Scheme of the construct for RNAi. The highly conserved coding region in *PVS1*–*PVS4* was used as a trigger for RNAi silencing (Fig. S1A,B). The conserved 488 bp region in *PVS3* was sub‐cloned into pHANNIBAL vector (Wesley et al., 2001). Then, the *Not*I‐digested fragment was sub‐cloned into a binary vector. (B) Transcript accumulation of *PVS* genes in response to hyphal wall components (HWC) elicitor 6 h after the treatment. Total RNAs were extracted from tubers and leaves from the wild‐type (WT) and two independent transgenic potato plants, RNAi‐17 (#17) and RNAi‐30 (#30). The transcript levels were determined by real time Reverse Transcription‐quantitative Polymerase Chain Reaction (RT‐qPCR) using specific primers for each *PVS* gene. Data are means ± standard deviations (SDs) from at least three experiments. Asterisks indicate statistically significant differences compared with WT (Student's *t*‐test, **P < 0.01). (C) Analysis of siRNAs from the tubers and the leaves using RNAs in (B). The bottom section shows rRNA as a loading control.

To investigate the role of phytoalexins in potato plants, we generated transgenic potato plants in which the *PVS* genes were silenced. Because cultivated potatoes have multiplex chromosomes, many allelic variations of isogene exist (Joos and Hahlbrock, 1992). Therefore, we adapted an RNAi strategy to silence all *PVS* genes in potato cultivar Sayaka, which is tetraploid (Fig. 2A). We used a highly conserved 488 bp coding region of *PVS3* as the trigger dsRNA (Fig. S1A,B). The relative nucleotide identities of the corresponding region between *PVS3* and the other three members of the gene family were 94%–95%. We transformed potato plants with the RNAi construct (Fig. 2A) and obtained transformants (RNAi‐17 and RNAi‐30) showing normal development of tubers and leaves similar to wild‐type plants (Fig. S2). We estimated the mRNA levels of *PVS1–PVS4* at 6 h after treatment of these transgenic plants with the hyphal wall components (HWC) elicitor of *P. infestans* (Doke and Tomiyama, 1980; Yoshioka *et al*., 2001) using real time Reverse Transcription‐quantitative Polymerase Chain Reaction (RT‐qPCR). The mRNA levels of *PVS1*–*PVS4* in tubers and leaves were highly suppressed in transgenic potato line RNAi‐17 and RNAi‐30 compared with the wild‐type potato (Fig. 2B). Notably, the expression level of *PVS3* in wild‐type leaves was much higher than for *PVS1, ‐2* and ‐*4*, suggesting that PVS3 seems to be a central player in the leaves in agreement with previous reports (Yamamizo *et al*., 2006; Yoshioka *et al*., 1999). We confirmed the accumulation of short interfering RNA (siRNA), a molecular marker for dsRNA‐based gene silencing, in HWC‐treated tubers and leaves of RNAi‐17 and ‐30 (Fig. 2C).

**Figure 3:**
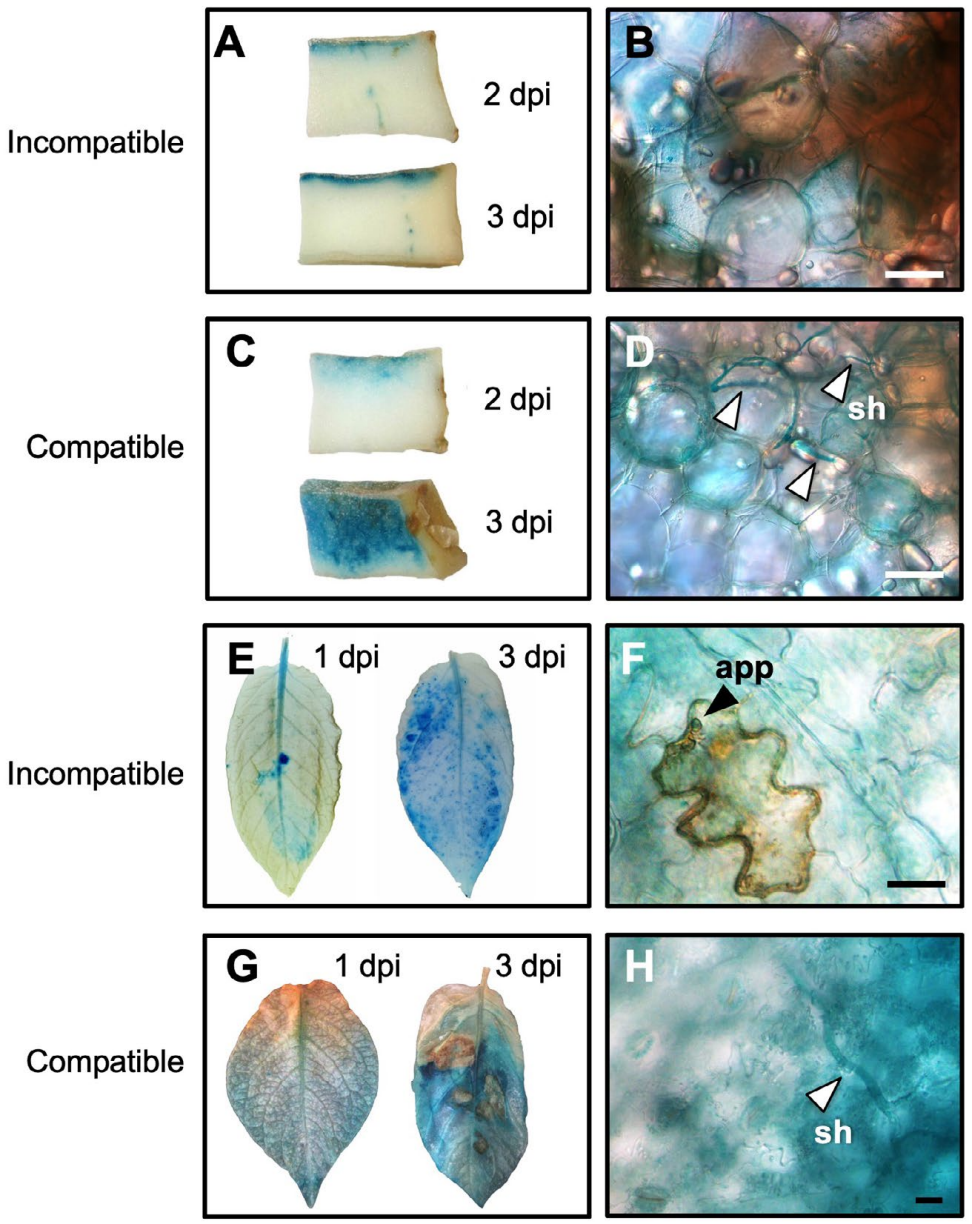
Expression profile of the *GUS* gene under the control of the *PVS3* promoter in transgenic potato (*Solanum tuberosum*) plants. The surfaces of tuber slices were inoculated with *Phytophthora infestans*, then cut vertically, stained and observed for GUS. GUS‐stained tubers inoculated with an avirulent (A) and a virulent isolate (C) of *P*. *infestans*. GUS‐stained tuber surfaces were horizontally sliced and were observed with a microscope at 2 days post‐inoculation (dpi) (B) and at 3 dpi (D). GUS activities in *PVS3p:GUS*‐expressed potato leaves after inoculation with an avirulent (E) or virulent isolate (G) of *P*. *infestans*. (F) Close‐up of the inoculated leaves from (E) at 1 dpi. (H) Close‐up of inoculated leaves from (G) at 3 dpi. White arrowheads: secondary hyphae (sh); black arrowhead: appressorium (app). Bars, 20 µM.

Previously we reported that transcript levels of *PVS* genes in tubers and leaves were transiently induced after inoculation with an avirulent or virulent isolate of *P*. *infestans* (Yamamizo *et al*., 2006; Yoshioka *et al*., 1999). To investigate the spatiotemporal expression profiles of the *PVS* gene in response to *P*. *infestans*, we tested the response of the *PVS3* promoter against pathogen infection using transgenic potato plants containing *PVS3p:GUS* (Fig. 3). The *PVS3* promoter in tubers did not respond to wounding treatment, because there was no GUS staining on the cut surface, and the promoter in leaves did not respond to water treatment (Fig. S3), as we reported previously (Yamamizo *et al*., 2006). Transgenic tuber slices were cut in a vertical direction, and histochemical localization of GUS activity *in situ* was monitored. In incompatible interactions, GUS activity was detected at the inoculated surface of tubers at 2 days after inoculation, and strong GUS staining was observed at 3 days after inoculation (Fig. 3A). This GUS‐stained area did not extend toward the opposite side of the tuber slice. When a transverse section of an inoculated tuber surface was observed with a microscope at 2 d after inoculation, HR cell death was observed, and GUS staining was seen around the dead cells (Fig. 3B). In compatible interactions, the GUS‐stained area extended gradually as secondary hyphae extensively colonized the tissue at 3 days after inoculation (Fig. 3C), when necrosis on tuber tissue was observed and strong GUS staining was seen around the secondary hyphae (Fig. 3D). Thus, the *PVS3* promoter was induced by both avirulent and virulent isolates of *P*. *infestans* in tubers. GUS activity was also detected in leaves inoculated with avirulent or virulent isolates of *P*. *infestans* 1 day after inoculation, and GUS activity was strong in the inoculated leaves at 3 days after inoculation (Fig. 3E,G). With incompatible interactions, GUS staining was seen in neighbouring cells of a dead epidermal cell invaded by the pathogen (Fig. 3F). Necrotic spots were visible in leaves at 3 days after inoculation with virulent *P*. *infestans*, and GUS staining was also seen around these spots (Fig. 3G). GUS activity was also very strong around secondary hyphae (Fig. 3H). These observations indicated that the *PVS3* gene is quickly induced not only by an avirulent, but also by virulent isolate of *P*. *infestans* in tubers and leaves. We similarly confirmed that *HMGR2*, another key gene for phytoalexin synthesis, was induced in response to avirulent *P*. *infestans* in leaves (Fig. S4).

**Figure 4:**
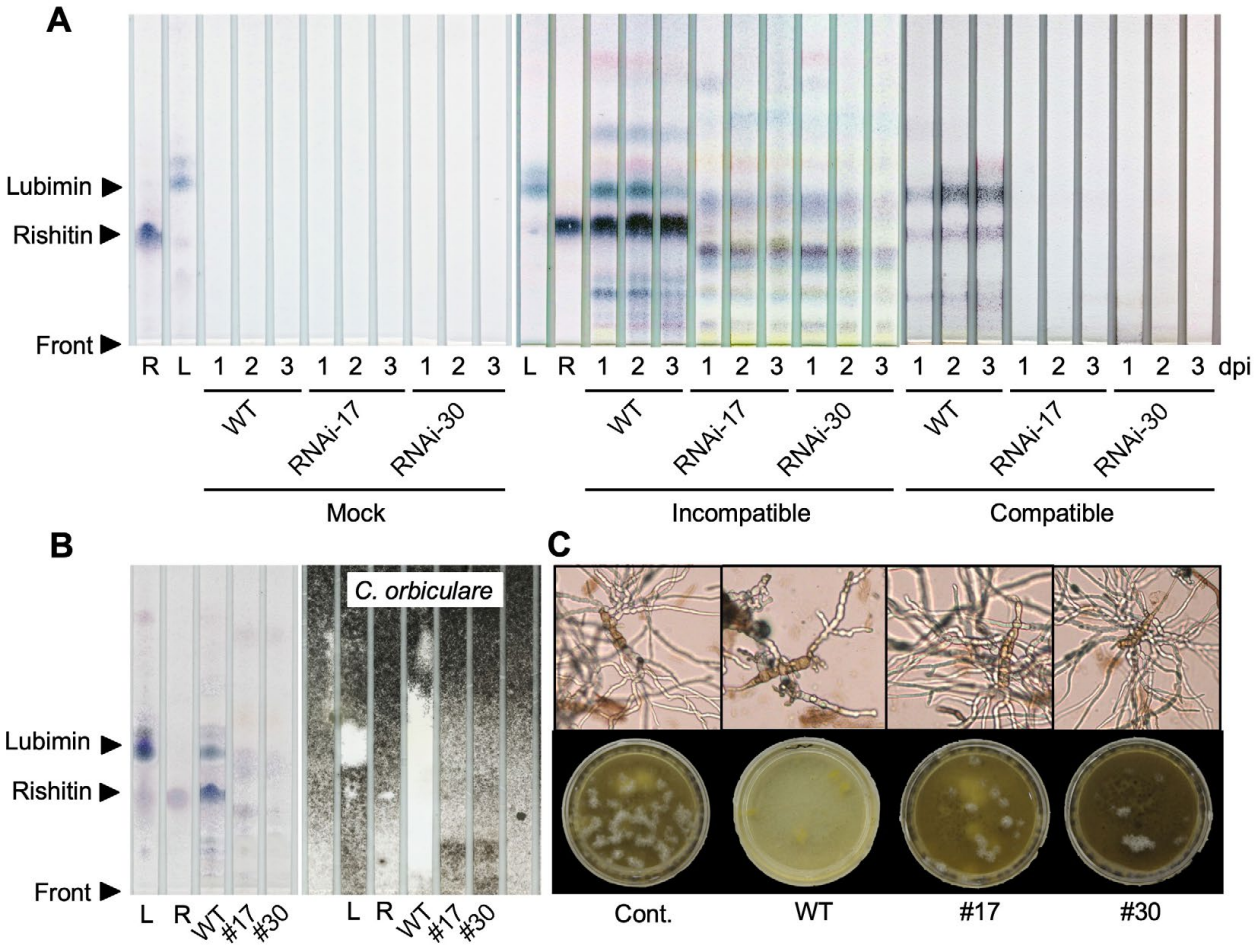
*PVS*‐silenced potato (*Solanum tuberosum*) tubers do not accumulate antimicrobial phytoalexins in response to *Phytophthora infestans*. (A) Potato tubers of wild‐type (WT), RNAi‐17 and RNAi‐30 were inoculated with an avirulent (incompatible interaction) or a virulent isolate (compatible interaction) of *P*. *infestans*. Phytoalexins were extracted at 1 day, 2 days and 3 days post‐inoculation (dpi). Crude phytoalexin extracts and 20 µg purified lubimin and rishitin were separated and developed on thin layer chromatography (TLC) plates. (B) Phytoalexins were extracted from tubers of the WT, RNAi‐17 (#17) and RNAi‐30 (#30) at 1 dpi with an avirulent isolate of *P*. *infestans*. Crude phytoalexin extracts were separated on TLC plates, then sprayed with a conidial suspension of *Colletotrichum orbiculare* to detect any antifungal activity. White spots indicate zones of antimicrobial activity. (C) Spores of *Alternaria solani* were incubated in liquid medium containing crude phytoalexin extracts from tubers of WT, #17 and #30 at 1 dpi with avirulent *P*. *infestans*. Methanol as a solvent was used as a control (Cont.). Upper panel: Light micrographs of cultures in plastic dishes at 1 dpi. Lower panel: Cultures in dishes at 7 dpi.

When potato tubers are inoculated with an avirulent isolate of *P*. *infestans*, rishitin and lubimin accumulate. Rishitin in potato tubers begins to accumulate within 6 h and reaches a maximum 3 days to 4 days after inoculation (Doke *et al*., 1996; Horikawa *et al*., 1976; Tomiyama *et al*., 1968). To investigate the effect of knockdown of the *PVS* genes on phytoalexin production in transgenic tubers, we placed zoospore suspensions of an avirulent or virulent isolate of *P*. *infestans* into holes in tuber slices. At 1 day, 2 days and 3 days after inoculation, the inoculation fluids were collected for phytoalexin extraction, and the extracts were separated on thin layer chromatography (TLC) plates. Rishitin and lubimin rapidly accumulated after inoculation with the avirulent isolate of *P*. *infestans* in wild‐type tuber slices but were not detected in the RNAi‐17 and ‐30 transgenic tubers (Fig. 4A). Other secondary metabolites, which have R_f_ values different from rishitin and lubimin, were detected in RNAi‐17 and ‐30 in the incompatible interactions (Fig. 4A). Rishitin and lubimin also slightly accumulated in wild‐type tuber slices in the compatible interactions (Fig. 4A). Contradictorily, *PVS* mRNA and PVS activities in the incompatible and compatible interactions in potato tubers are induced at similar levels (Zook and Kuć, 1991). Here, we also detected GUS activities in tubers and leaves of *PVS3p:GUS* transgenic plants in both interactions (Fig. 3). However, activity of the HMGR enzyme, another key enzyme for phytoalexin synthesis, is high in tubers inoculated with avirulent *P*. *infestans* compared with virulent *P*. *infestans* (Yoshioka *et al*., 1996). Thus, the weaker phytoalexin accumulation in the compatible interaction might be due to differential regulation of HMGR.

The metabolites extracted from tubers inoculated with the avirulent isolate of *P*. *infestans* 1 day after inoculation were then analysed by high performance liquid chromatography (HPLC) (Fig. S5). Rishitin and lubimin peaks were detected in extracts from wild‐type tubers, but not from RNAi‐30 tubers. Instead, unknown hydrophilic metabolites were detected at a retention time of 15 min–17.5 min in fractions from RNAi‐30 tubers (Fig. S5). Thus, rishitin and lubimin accumulation was not observed in *PVS*‐silenced transgenic potato tubers during *P*. *infestans* infection. These transiently induced, unknown products in transgenic tubers after inoculation of avirulent *P*. *infestans* were then tested for antifungal activity. The extracts were separated on a TLC plate, and a conidial suspension of *C*. *orbiculare* was sprayed on the plate. White spots that indicate zones of antifungal activity were observed on the extracts from the wild‐type tuber, but not from RNAi‐17 and ‐30 (Fig. 4B). Moreover, an inhibition ring assay indicated that the metabolites from tubers of RNAi‐17 and ‐30 did not inhibit hyphal growth of *P*. *infestans* (Fig. S6). We also tested antifungal activity against *A*. *solani* by co‐incubating a spore suspension with the extracts. Only extracts from the wild‐type tuber were inhibitory to growth of *A*. *solani* (Fig. 4C). These results suggest that *PVS*‐silenced tubers do not produce phytoalexins after *P*. *infestans* infection.

### Phytoalexins contribute to tuber resistance against *P*. *infestans* infection

**Figure 5:**
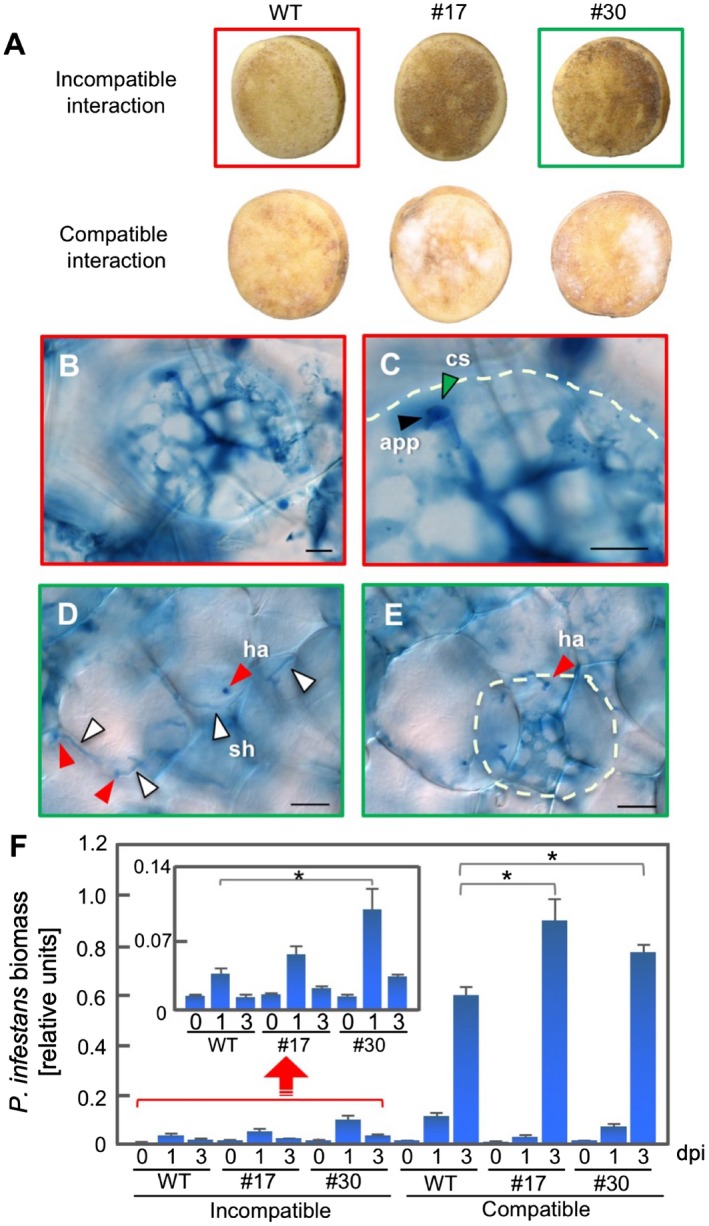
Effects of *PVS*‐silencing on susceptibility of potato (*Solanum tuberosum*) tubers to *Phytophthora infestans*. (A) Tubers of wild‐type (WT), RNAi‐17 (#17) and RNAi‐30 (#30) were inoculated with an avirulent (incompatible interaction) or virulent isolate (compatible interaction). For the incompatible interactions, inoculated tuber surfaces were photographed 2 days after inoculation (dpi). For compatible interactions, the opposite side of inoculated tubers was photographed at 4 dpi. B–E: Light micrographs of tuber slices stained with trypan blue, viewed with differential interference contrast optics. (B) WT tuber surface inoculated with avirulent isolate (in red box in A). Bar, 20 µM. (C) Enlarged image of (B). The dotted line shows cell undergoing hypersensitive response (HR) cell death. Bar, 20 µM. (D) RNAi‐30 tuber surface inoculated with avirulent isolate (in green box in A). Bar, 50 µM. (E) Image of cells below the penetrated cell in (D). The dotted line indicates a cell undergoing HR cell death. Bar, 50 µM. Black arrowhead, appressorium (app); green arrowhead, cystospore (cs); red arrowhead, haustoria (ha); white arrowhead, secondary hyphae (sh). (F) Determination of *P*. *infestans* biomass by real‐time quantitative Polymerase Chain Reaction (PCR) with *P*. *infestans*‐specific primers using DNA isolated from inoculated tubers. Biomass was determined at 0 dpi, 1 dpi and 3 dpi. Inset: rescaled graph for the incompatible interaction. Data are means ± standard deviations (SDs) from three experiments. Data were analysed by Student's *t*‐test: *, *P* < 0.05 versus WT tubers.

In *P*. *infestans*–potato interactions, both avirulent and virulent isolates can penetrate and produce an infection vesicle in the first attacked cell, but HR cell death is induced only in the incompatible interaction. In the compatible interaction, *P*. *infestans* develops branching secondary hyphae in the intercellular space and forms haustoria in neighbouring cells (Kamoun *et al*., 1999; Tomiyama, 1956). To investigate the role of the phytoalexins during infection of tubers, we inoculated *PVS*‐silenced tubers with an avirulent or virulent isolate of *P*. *infestans* and also inoculated wild‐type tubers with the pathogens as a control (Fig. 5A). In the incompatible interactions, HR cell death was observed on the cut surface of the wild‐type tuber at 2 days after inoculation. Unexpectedly, massive HR cell death occurred over the entire surface of RNAi‐17 and RNAi‐30 tubers. In the compatible interactions, aerial mycelia were observed on the opposite side of inoculated tuber surfaces of RNAi‐17 and ‐30 at 4 days after inoculation, while much less mycelial growth was found on the wild‐type tubers (Fig. 5A).

We then used trypan blue to stain the sliced surfaces of tubers 1 day after inoculation and observed the early infection process with a microscope. In the first layer of the wild‐type tuber surface, the cytoplasm in the cell penetrated by avirulent *P*. *infestans* had aggregated and was undergoing HR cell death (Fig. 5B,C). In contrast to invaded cells of the wild‐type, invaded cells of *PVS*‐silenced tubers in the incompatible interactions had secondary hyphae with haustoria (Fig. 5D), suggesting that post‐invasive resistance associated with HR cell death could be involved in PVS‐mediated immune responses. In the next layer of cells below a cell with a haustorium, the cytoplasm had aggregated (Fig. 5E). We speculate that massive HR cell death seen in *PVS*‐silenced tubers in Fig. 5A may be a consequence of the extensive HR cell death in the second layer of cells, and robust HR cell death blocks further infection, showing a trailing necrosis‐like phenotype (Uknes *et al*., 1992).

Pathogen biomass in inoculated tubers was determined by qPCR (Asai *et al*., 2008; Ishihama *et al*., 2011). In the incompatible interactions, pathogen biomass in RNAi‐17 and ‐30 was higher than in wild‐type tubers at 2 days after inoculation (Fig. 5F, inset). Subsequently, pathogen biomass decreased to a level similar to wild‐type tubers at 3 days after inoculation (Fig. 5F, inset), suggesting that secondary hyphae in the first attacked cells and intercellular space had collapsed and that their genomic DNA was degraded by immune responses by that time. In the compatible interactions, the pathogen biomass in RNAi‐17 and in RNAi‐30 had significantly increased by 3 days after inoculation compared with those in the wild‐type tubers (Fig. 5F). These results indicate that deficient phytoalexin production in tubers affects resistance against *P*. *infestans* in the incompatible interaction and susceptibility in the compatible interaction.

### *PVS*‐silenced leaves are more susceptible than the wild‐type to *P*. *infestans*

**Figure 6:**
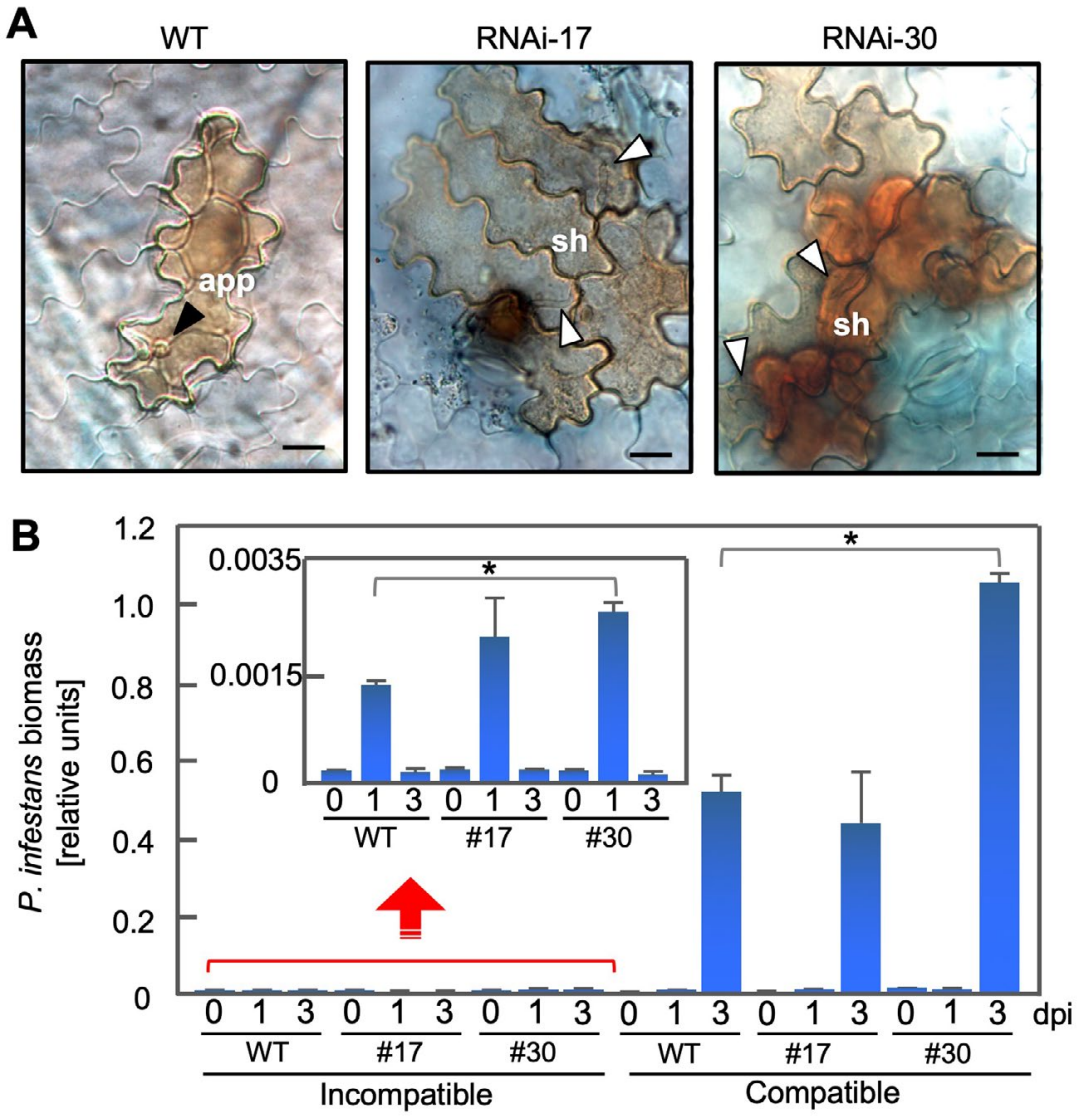
*PVS*‐silenced potato (*Solanum tuberosum*) leaves are more susceptible than the wild‐type (WT) to avirulent and virulent *Phytophthora infestans*. (A) Light micrographs of WT and transgenic potato leaves (RNAi‐17 and RNAi‐30) were inoculated with an avirulent isolate of *P*. *infestans*. Leaves were stained with trypan blue and observed using differential interference contrast optics. Black arrowhead (WT): appressorium (app); white arrowheads: secondary hyphae (sh), respectively. Bars, 20 µM. (B) Determination of *P*. *infestans* biomass by real‐time quantitative Polymerase Chain Reaction (PCR) with *P*. *infestans*‐specific primers using DNA isolated from inoculated leaves of WT, RNAi‐17 (#17) and RNAi‐30 (#30), respectively. Biomass was determined at 0 days, 1 day and 3 days post‐inoculation (dpi). Inset: rescaled graph in the incompatible interaction. Data are means ± standard deviations (SDs) from three experiments. Data were analysed by Student's *t*‐test; *P < 0.05 versus WT leaves.

To investigate the effect of the knockdown of *PVS* on pathogen development in leaves, we inoculated wild‐type and transgenic potato leaves with an avirulent isolate of *P*. *infestans*. Inoculated tissues were stained with trypan blue and observed with a microscope at 3 days after inoculation (Fig. 6A). In wild‐type leaves, browned cells, which resulted from HR cell death, were observed in the attacked epidermal cell, but secondary hyphae were observed inside epidermal cells of RNAi‐17 and ‐30 even though the isolate was avirulent. In addition to the HR cell death in the attacked epidermal cells, massive HR cell death was observed in mesophyll cells below the penetrated epidermal cells (Fig. 6A; RNAi‐30). The qPCR to determine the biomass of avirulent and virulent isolates of *P*. *infestans* in inoculated leaves (Fig. 6B) showed that, in the incompatible interactions, pathogen biomass in RNAi‐17 and ‐30 leaves was higher than in wild‐type leaves at 1 day after inoculation. By 3 days after inoculation, biomass had decreased to a level comparable to that in wild‐type leaves (Fig. 6B, inset), similar to the case in tubers (Fig. 5F, inset). In the compatible interactions, pathogen biomass had drastically increased by 3 days after inoculation, but greater biomass was detected in RNAi‐30 leaves than in the wild‐type (Fig. 6B). *PVS*‐silenced leaves were more susceptible than the wild‐type to *P*. *infestans*. As shown in the *PVS‐*silenced tubers (Fig. 5D,E), avirulent isolates were able to infect leaf epidermal cells, even in the incompatible combinations (Fig. 6A).

When extracted metabolites from the wild‐type or RNAi‐30 leaves inoculated with avirulent *P*. *infestans* at 1 day after inoculation were separated on a TLC plate or analysed by HPLC, rishitin and lubimin were not detected (Fig. S7A,B). Thus, rishitin and lubimin did not accumulate in leaves after *P*. *infestans* infection, agreeing with the report of Rohwer *et al*. (1987). These results suggest that *PVS* in leaves participates in post‐invasive defence against *P*. *infestans*.

### *PVS*‐silenced leaves were more susceptible than the wild‐type to *A*. *solani*

**Figure 7:**
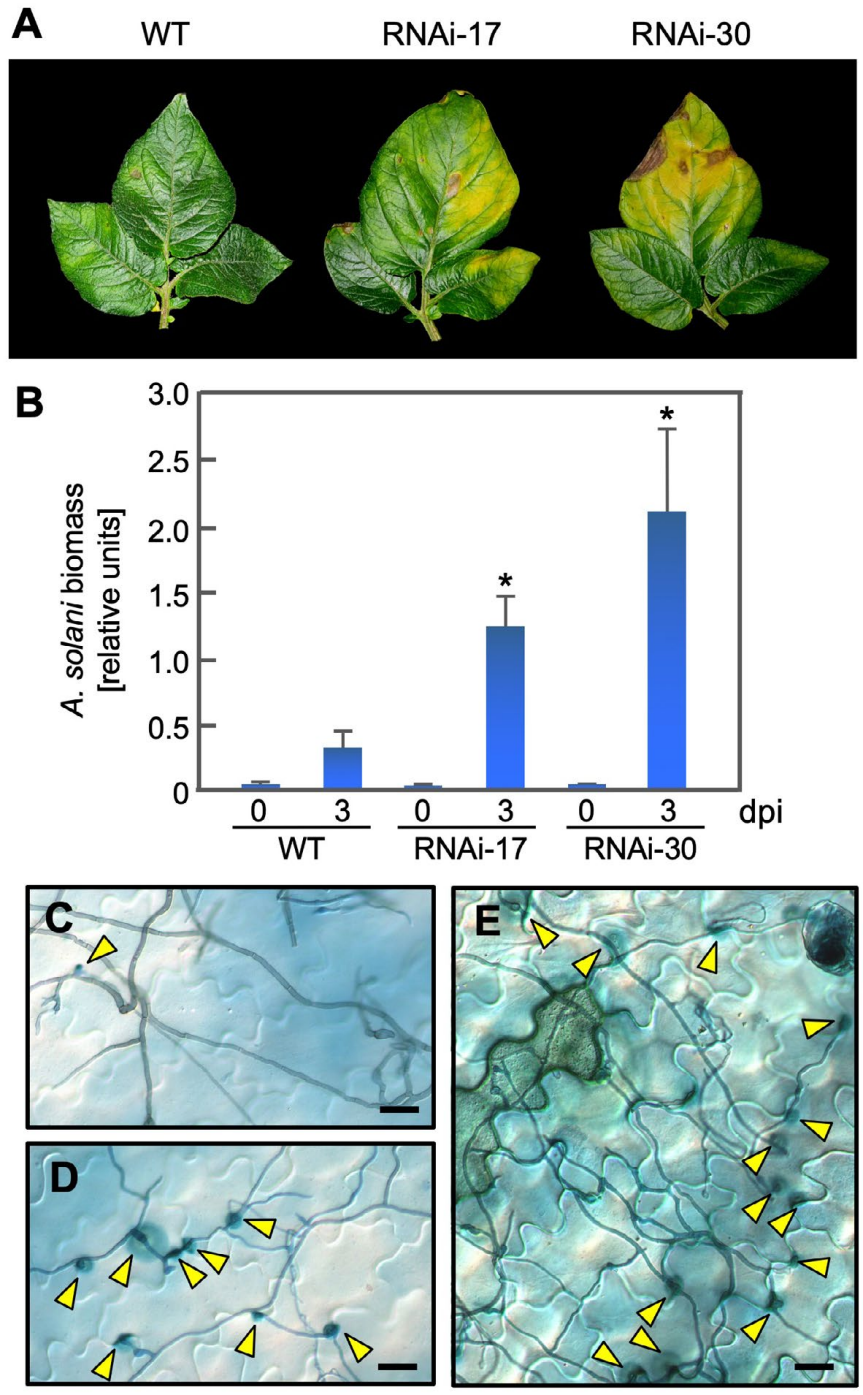
*PVS*‐silenced potato (*Solanum tuberosum*) leaves are more susceptible than the wild‐type (WT) to *Alternaria solani*. (A) 7 days post‐inoculation (dpi) of WT and transgenic potato leaves (RNAi‐17 and RNAi‐30) with *A*. *solani*. (B) Determination of *A*. *solani* biomass by real time quantitative Polymerase Chain Reaction (PCR) with *A*. *solani*‐specific primers using DNA isolated from inoculated leaves. Biomass was determined at 0 dpi and 3 dpi. Data are means ± standard deviations (SDs) from three experiments. Data were analysed by Student's *t*‐test: *, *P* < 0.05 versus wild‐type leaves. (C) Wild‐type, (D) RNAi‐17 and (E) RNAi‐30 leaves were stained with trypan blue solution 3 dpi and were observed with a microscope. Yellow arrowheads: penetration sites. Bars, 20 µM.

Because camalexin inhibits growth of *A*. *brassicicola* in *A*. *thaliana* (Thomma *et al*., 1999), we investigated effects of *PVS*‐silencing on resistance to the potato early blight necrotrophic pathogen, *A*. *solani* in wild‐type and transgenic potato leaves. Disease symptoms were more severe on RNAi‐17 and ‐30 leaves than the wild‐type at 7 days after inoculation (Fig. 7A). Determination of *A*. *solani* biomass by qPCR showed that the pathogen biomass was much higher in RNAi‐17 and ‐30 leaves than in wild‐type leaves (Fig. 7B). Observations of trypan‐blue‐stained inoculated leaves 3 days after inoculation (Fig. 7C–E) showed frequent penetration of epidermal cells and extensive fungal hyphae in RNAi‐17 and ‐30 leaves (Fig. 7D,E) compared to wild‐type leaves (Fig. 7C). These results suggest that PVS is involved in pre‐invasive defence against necrotrophic pathogens, presumably through unidentified sesquiterpenoid compounds. Germ tubes from zoospores of *P*. *infestans* penetrate potato cells 2 h to 3 h after inoculation (Yoshioka *et al*., 1996), while those from spores of *A*. *solani* penetrate around 24 h after inoculation (Kobayashi *et al*., 2012). Although *A*. *solani* might be attacked by PVS‐mediated compounds before penetration, these adapted pathogens are thought to have a detoxification system for the host antimicrobial compounds (Giannakopoulou *et al*., 2014).

## Discussion

To cope with diverse pathogen attacks in natural environments, plants have evolved a diverse system of innate immunity against pathogens (Jones and Dangl, 2006; Macho and Zipfel, 2014). Production of antimicrobial compounds is part of the central immune system. To defend against various pathogens, plants constitutively store antimicrobial compounds termed phytoanticipins, such as saponins in oats (Osbourn *et al*., 2011) and glucosinolates, cyanogenic glucosides and benzoxazinone glucosids in *A*. *thaliana* (Frey *et al*., 2009; Halkier and Gershenzon, 2006; Møller, 2010). On the other hand, phytoalexins are newly synthesized secondary metabolites against pathogen invasion that generally have strong antimicrobial activity (Ahuja *et al*., 2012; Piasecka *et al*., 2015). Sorghum, that lacks a myeloblastosis (MYB) transcription factor regulating biosynthesis of 3‐deoxyanthocyanidins, including apigeninidin and luteolinidin, does not produce 3‐deoxyanthocyanidins when challenged by a pathogen and is more susceptible to *Colletotrichum sublineolum* (Ibraheem *et al*., 2010). This study showed that 3‐deoxyanthocyanidins have a central role in the immune response to anthracnose fungi. It also has been demonstrated that sesquiterpenoid phytoalexins produced by solanaceous plants are correlated with resistance to *P*. *infestans*. Virus‐induced gene silencing of *EAS* compromises capsidiol and related phytoalexin production in *N*. *benthamiana* leaves, and *P*. *infestans* successfully infects and fully develops inside silenced leaves (Shibata *et al*., 2016). Likewise, silencing of *EAS* in pepper by a virus vector results in a significant reduction of capsidiol accumulation and growth of non‐adapted *P*. *infestans* (Lee *et al*., 2017). Such correlations have also been predicted for potato–*P*. *infestans* interactions (Choi *et al*., 1992; Sato *et al*., 1971; Tomiyama *et al*., 1968; Yoshioka *et al*., 1996; Zook and Kuć, 1991). Therefore, we expected that the potato phytoalexins lubimin and rishitin also have pivotal roles in resistance to potato blight pathogens *P*. *infestans* and *A*. *solani*. However, there has been no genetic evidence for the roles of lubimin and rishitin in defence responses to these pathogens.

### Potato phytoalexins are required for post‐invasive resistance or HR cell death to *P*. *infestans* in tubers

In this study, we used *PVS*‐RNAi to generate phytoalexin‐deficient potato plants and inoculated tubers with a virulent isolate of *P*. *infestans* (Fig. 5A). Aerial mycelia on tubers of RNAi‐17 and ‐30 in compatible interactions suggested that a deficiency in rishitin and lubimin allowed hyphal growth of the pathogen. Although the phytoalexins were detected in both the incompatible and compatible interactions (Fig. 4A), rishitin accumulated much faster and at much higher levels in the incompatible interactions. We previously showed that the *PVS3* promoter was controlled by SIPK/WIPK in *N*. *benthamiana* leaves (Yamamizo *et al*., 2006). In the present study, the *PVS3* promoter was activated in both incompatible and compatible interactions in the tubers and leaves (Fig. 3), and Zook and Kuć (1991) reported that PVS enzymatic activities were activated at similar levels in both interactions. On the other hand, HMGR activity in tubers increases rapidly during the incompatible but not in the compatible interaction (Yoshioka *et al*., 1996), suggesting that this may result in high rishitin accumulation in response to the avirulent isolate of *P*. *infestans*. However, this avirulent isolate could not cause disease symptoms on tubers of RNAi‐17 and ‐30, suggesting that lubimin and rishitin have only a marginal role in resistance of potato to *P*. *infestans* or that ETI‐mediated multiple defence responses block pathogen growth. Capsidiol is more effective against the non‐adapted potato pathogen *P*. *infestans* than against the adapted pepper pathogen *P*. *capsici* (Giannakopoulou *et al*., 2014; Lee *et al*., 2017), suggesting that adapted pathogens have developed a system to tolerate the phytoalexins produced by their hosts.

ETI responses are often accompanied by HR cell death, implying that phytoalexins concentrate by influx into dead cells from surrounding cells to kill the pathogens (Sato *et al*., 1971). Microscopic observation indicated that an avirulent isolate of *P*. *infestans* formed secondary hyphae and haustoria in the first attacked cell of RNAi‐30 tuber 1 day after inoculation (Fig. 5D), even though HR cell death was induced in the first attacked cell of wild‐type tuber (Fig. 5B,C). At this time in the incompatible *P*. *infestans*–potato interaction, lubimin and rishitin levels are very high (Fig. 4A). These results suggested that suppression of phytoalexin‐mediated post‐invasive defence in RNAi‐30 tubers might enable the pathogen to form infection structures in cells with ongoing HR. Alternatively, reduced production of lubimin and rishitin might result in suppression or delay of HR cell death during potato ETI. RNAi‐mediated silencing of key enzymes for the synthesis of glyceollins, soybean isoflavonoid phytoalexins, suppresses resistance to an avirulent isolate of oomycete pathogen *Phytophthora sojae* and HR cell death (Graham *et al*., 2007). Taken together, these results suggest a correlation between pathogen‐triggered accumulation of antimicrobial phytoalexins and HR cell death, although we currently do not know the molecular mechanisms.

### Effects of *PVS*‐silencing on immune responses in potato leaves

Because inoculation assays of *PVS*‐silenced leaves were more susceptible than the wild‐type to *P*. *infestans* and *A*. *solani*, we tested extracts from leaves, excluding petioles and midribs, and found no lubimin and rishitin using TLC or HPLC (Fig. S7), and there were no differences in the chromatograms of extracts from wild‐type and *PVS*‐silenced leaves. We also performed an inhibition ring assay for extracted metabolites from leaves, but none of the samples, including extracts from wild‐type leaves, inhibited mycelial growth of *P*. *infestans* (Fig. S8). Sesquiterpenoid phytoalexins do not accumulate detectable amounts in potato leaves (Rohwer *et al*., 1987). By contrast, four phytoalexins, solavetivone, phytuberin, lubimin and rishitin (Fig. 1), were detected in potato leaves infected by *P*. *infestans* using TLC (Andreu *et al*., 2001). However, the possibility that potato leaf samples used in the experiment may contain petioles and midribs, which potentially produce phytoalexins (Sato *et al*., 1971), cannot be excluded. In addition, a reverse‐phase HPLC study of secondary metabolites in leaves of two potato cultivars after infection with two isolates of *P*. *infestans* (Henriquez *et al*., 2012) showed that the field resistant potato cultivar produces an unidentified terpenoid, probably conferring resistance to the tested isolates.

We believe that the PVS‐mediated compounds in leaves have pivotal roles in immune responses, even though we could not confirm the existence of phytoalexins for the following reasons: (i) *PVS*‐silenced leaves were more susceptible to late and early blight pathogens (Figs 6 and 7), (ii) two key genes for phytoalexin synthesis, *HMGR2* and *PVS3*, in leaves were induced after inoculation with the avirulent isolate of *P*. *infestans* (Figs 2 and S4), (iii) detection of phytoalexins in leaves is difficult because of their rapid degradation or low levels, where the level of sesquiterpenoid phytoalexin accumulation might depend on the potato cultivar, as suggested by Andreu *et al*. (2001), (iv) we cannot rule out the possibility that PVS mediates production of certain volatile compounds, because the volatile sesquiterpene (*E*)‐β‐caryophyllene directly inhibits pathogen growth in *A*. *thaliana* (Huang *et al*., 2012).

### PVS participates in pre‐invasive resistance to *A*. *solani* and post‐invasive resistance to *P*. *infestans*

Pathogen sensing by non‐host plants also triggers phytoalexin accumulation similar to the case for host resistance. Asian soybean rust pathogen *Phakopsora pachyrhizi* induces cell death in penetrated epidermal cells of alfalfa and elicits medicarpin phytoalexin production that inhibits urediniospore germination and differentiation (Ishiga *et al*., 2015). In interactions between Arabidopsis and the non‐adapted hemibiotroph *Colletotrichum gloeosporioides*, tryptophan‐derived indole glucosinolates confer pre‐invasive resistance, and camalexin is involved in post‐invasive resistance by restricting subsequent pathogen development and spread to neighbouring cells (Hiruma *et al*., 2013). Capsidiol production in pepper leaves seems to confer post‐invasive resistance to non‐adapted *P*. *infestans* (Lee *et al*., 2017). Thus, antimicrobial compounds are likely to be common to host and non‐host resistance in various pathogen–plant interactions. The immune response to non‐adapted pathogens is thought to be triggered by a combination of PRRs and NLRs, although the exact mechanism is not known (Schulze‐Lefert and Panstruga, 2011).

In the present study, *PVS*‐silenced potato leaves inoculated with *A*. *solani* showed severe disease symptoms accompanied by increased penetration rates (Fig. 7), suggesting that PVS‐mediated compounds confer pre‐invasive resistance to the necrotrophic pathogen. In *A*. *solani*–potato interactions, immune responses may be attributed to the PTI response via PAMP recognition, which might be suppressed by a phytotoxin, alternaric acid (Langsdorf *et al*., 1991). AAL‐toxin, which is produced by necrotrophic pathogen *Alternaria alternata* f. sp. *lycopersici*, is a pathogenicity factor induces cell death in its sensitive natural host tomato and in some *Nicotiana* spp. (Brandwagt *et al*., 2001; Wang *et al*., 1996). Microarray analysis indicated that AAL‐toxin provokes cell death with less up‐regulation of defence‐related genes (Gechev *et al*., 2004; Mase *et al*., 2013), suggesting that the phytotoxin appears to hijack the plant immune system to induce cell death and subsequent successful infection. We previously showed that *A*. *solani* causes more severe symptoms on the transgenic potato plants, which activate reactive oxygen species (ROS)‐generating NADPH oxidase in response to pathogen attack, than on the wild‐type while the transgenic plants are more resistant to *P*. *infestans* (Kobayashi *et al*., 2012). Multiple lines of evidence suggest that pre‐invasive chemical barriers can block necrotrophic pathogens, which absorb nutrients from dead cells. On the other hand, here we found that lubimin and rishitin in tubers are involved in the ETI‐triggered HR in response to *P*. *infestans* (Fig. 5). *PVS*‐silenced potato leaves showed the trailing cell death‐like phenotype against avirulent *P*. *infestans* (Fig. 6), suggesting that PVS‐mediated compounds function in post‐invasive resistance. Thus, the relative contribution of antimicrobial components to the mode of defence may vary depending on the pathogen species, suggesting that PVS‐mediated compounds participate in pre‐invasive resistance to *A*. *solani* and post‐invasive resistance to *P*. *infestans*. Identification of PVS‐mediated compounds in potato leaves induced by pathogen invasion remains to be further investigated.

## Experimental Procedures

### Plant growth conditions

Potato plants (*Solanum tuberosum*) were grown in a biotron at 20 °C, 70% humidity with 16 h light/8 h dark.

### Pathogen inoculation

*P*. *infestans* races 0 and 1.2.3.4 were maintained on susceptible potato tubers, and suspensions of *Phytophthora* zoospores were prepared as described previously (Yoshioka *et al*., 2003). A zoospore suspension (1 × 10^5^ zoospores/mL) was applied to leaves on potato plants or aged tuber slices by using lens paper to disperse the zoospores under high humidity at 20 °C.

*A*. *solani* was grown on oatmeal agar for 7 days, then aerial mycelia were rubbed off using wet cotton balls. The remaining mycelia were exposed to black and blue light at 25 °C for 4 days to induce sporulation. The produced spores were suspended in water at 5 × 10^5^ spores/mL. For determination of *A*. *solani* biomass, 5 µL drops of spore suspension were placed on detached potato leaves. For microscopic observation, a spore suspension was sprayed on leaves using an airbrush.

### Treatment of potato tuber discs and leaves with HWC

HWC were prepared from mycelia of *P*. *infestans* grown in liquid medium for 13 days at 20 °C as described previously (Doke and Tomiyama, 1980; Yoshioka *et al*., 2001). Aged potato tuber discs for 24 h were treated with 1 mg/mL HWC and incubated for indicated times in a moist chamber at 20 °C in the dark. Leaves were infiltrated with 0.5 mg/mL HWC and incubated for indicated times. Treated tuber discs and leaves were sampled for RNA extraction.

### Generation of transgenic plants

Potato plants (cv. Sayaka carrying *R1* and *R3*) were transformed with *PVS3p*: *β*‐*glucuronidase* (*GUS*) or *35S:PVS3‐RNAi* constructs. Generation of *PVS3p:GUS* transgenic plants was described previously (Yamamizo *et al*., 2006). For *35S:PVS3‐RNAi* transgenic plants, the following primers were used to amplify *PVS3* cDNA fragments. Restriction sites were added to the 5′ ends of the forward and reverse primer for cloning into pHANNIBAL vector (Wesley *et al*., 2001); antisense‐PVS3*‐Xho*I‐F (5′‐CCGCTCGAGGACCTCAAGTTCTTTTACTAT‐3′) and antisense‐PVS3*‐Eco*RI‐R (5′‐CGGAATTCAAGCTTCACATGTAAGGACTC‐3′), sense‐PVS3‐*Cla*I‐F (5′‐CCATCGATAAGCTTCACATGTAAGGACTCA‐3′) and sense‐PVS3‐*Bam*HI‐R (5′‐CGGGATCCGACCTCAAGTTCTTTTACTATT‐3′) (restriction sites are underlined), which produced 488 bp fragments. A construct made in pHANNIBAL was sub‐cloned as *Not*I fragment into pGeen0029 vector (Hellens *et al*., 2000), then was introduced into *Agrobacterium* strain LBA4404 by electroporation. Stable transgenic lines were generated by using *Agrobacterium*‐mediated gene transfer (Kobayashi *et al*., 2012). Independent transformed plant pools were kept separate for the selection of independent transgenic lines based on their kanamycin resistance.

### RNA isolation and real time RT‐qPCR

Total RNA from potato tubers and leaves were prepared using TRIzol reagent (Invitrogen, Carlsbad, CA, USA) according to the manufacturer's procedure. Reverse transcription was done using ReverTra Ace^R^ (TOYOBO CO., LTD, Osaka, Japan), and real time RT‐qPCR analysis was done using the StepOnePlus Real‐Time PCR system (Applied Biosystems, Foster City, CA, USA) with POWER SYBR GREEN PCR MASTER MIX (Applied Biosystems, Foster City, CA, USA). *EF‐1α* was used as a control in *S*. *tuberosum*. Table S1 lists the gene‐specific primers for each sequence.

### Detection of siRNA

*PVS3*‐derived siRNA was confirmed as described previously (Sunkar *et al*., 2005). Total RNA (10 µg per lane) was resolved on a denaturing 15% polyacrylamide gel and transferred electrophoretically to Hybond^TM^‐N^+^ (Amersham, Piscataway, NJ, USA) membranes. The membranes were ultraviolet (UV) cross‐linked and baked for 1 h at 80 °C. The cDNA probe was labelled with ^32^P‐dCTP using a random‐primed DNA labelling kit (Takara, Kusatsu, Japan). The probe was a 488 bp sense‐fragment of *PVS3* cDNA, which was amplified using primers sense‐PVS3‐*Cla*I‐F (5′‐CCATCGATAAGCTTCACATGTAAGGACTCA‐3′) and sense‐PVS3‐*Bam*HI‐R (5′‐CGGGATCCGACCTCAAGTTCTTTTACTATT‐3′) (restriction sites are underlined). Blots were pre‐hybridized for at least 10 min, then hybridized overnight using PerfectHYB Plus buffer (Sigma, St. Louis, MO, USA) at 30 °C. The blots were washed four times with 4 × SSC (SSC: 0.15 M NaCl and 0.015 M sodium citrate) and 0.1% v/v sodium dodecyl sulphate (SDS) for 5 min at 40 °C.

### GUS staining

Histochemical localization of GUS activity *in situ* was done using vacuum infiltration with a solution consisting of 50 mM sodium phosphate and 0.5 mg of 5‐bromo‐4‐chloro‐3‐indolyl glucuronide/mL. Leaves were vacuum‐infiltrated with the mixture for 16 h at 37 °C, then de‐stained in ethanol–acetic acid (3:1) overnight. Tuber tissues were incubated in the mixture for 5.5 h at 25 °C and used without de‐staining.

### Trypan blue staining

For visualizing cell death and fungal hyphal structures, leaves infected with *P*. *infestans* were transferred to a trypan blue solution (10 mL lactic acid, 10 mL glycerol, 10 g phenol, 10 mL H_2_O and 10 mg trypan blue) diluted in ethanol 1:1, then boiled for 3 h to 4 h. The leaves were then de‐stained overnight in 2.5 g/mL chloral hydrate. Stained leaves were observed using differential interference contrast optics (Axio Imager microscope, Carl Zeiss, Oberkochen, Germany).

### Extraction of phytoalexins

Phytoalexins that exuded from a *P*.*‐infestans*‐inoculated well bored in potato tubers into the surrounding tissues were extracted with ethyl acetate as described previously (Horikawa *et al*., 1976). *P*. *infestans*‐inoculated potato leaves were ground in liquid N_2_, then 50% methanol was added to extract soluble metabolites. The mixture was centrifuged, and phytoalexins were extracted from the supernatant using hexane–ethyl acetate (1:1) as described by Matsukawa *et al*. (2013). These crude phytoalexin extracts from tubers and leaves were then vacuum‐dried.

### Detection of phytoalexins by TLC

Extracted phytoalexins were dissolved in methanol, then separated on TLC plates (silica gel 60, Whatman, Maidstone, UK), which were developed with cyclohexane‐ethyl acetate (1:1) and visualized by spraying with sulfuric acid containing 0.5% vanillin followed by heating at 120 °C.

### HPLC analysis

The dried crude extracts from tubers or leaves were dissolved in acetonitrile and analysed using an Agilent 1100 series HPLC system (Agilent Technologies, Santa Clara, California, USA) with a Presto FF‐C18 (4.6 × 250 mM, Imtakt, Portland, Oregon, USA) column, Solvent A: distilled water; Solvent B: acetonitrile; flow: 0.15 mL/min; UV detection at 210 nM. Figures S3 and S5b show the gradient programmes.

### Evaluation of antifungal activity of extracted metabolites

For testing antifungal activity against *Colletotrichum orbiculare*, 100 µg of the dried extract and 20 µg purified lubimin and rishitin were separated on a TLC plate as described above, and the resultant plates were sprayed with a conidial suspension of *C*. *orbiculare* (1 × 10^7^ conidia/mL). The conidia were in a spore stock solution containing 0.7% potassium dihydrogen phosphate, 0.4% potassium nitrate, 0.3% sodium hydrogen phosphate, 0.1% magnesium sulphate, 0.1% sodium chloride and 5.0% glucose. The plates were then incubated at 25 °C under 100% humidity in the dark for a week. To test antifungal activity against *A*. *solani*, 2.5 mL of spore suspension (1 × 10^4^ spores/mL) in 1/5 Difco potato dextrose broth was incubated with 200 µg extracts at 25 °C in the dark for 2 weeks.

### Determination of *P*. *infestans* and *A*. *solani* biomass by qPCR

Biomass of *P*. *infestans* in three inoculated potato leaves was determined using qPCR and the method of Asai *et al*. (2008). *P*. *infestans*‐ (Judelson and Tooley, 2000) and plant‐specific DNA sequences were amplified using primers O8‐3 (5′‐GAAAGGCATAGAAGGTAGA‐3′) and O8‐4 (5′‐TAACCGACCAAGTAGTAAA‐3′) for *P*. *infestans* and StEF‐1α‐F (5′‐GGTCTACCAACCTCGACTGGTAC‐3′) and StEF‐1α‐R (5′‐GGGTTTGTCTGATGGCCTCTTGG‐3′) for potato plants. Biomass of *A*. *solani* in five potato leaf discs containing 5 µL of a conidial suspension (5 × 10^5^ spores/mL) was determined using qPCR as described by Kobayashi *et al*. (2012). The *A*. *solani*‐specific DNA sequence was amplified using primers Tubulin‐F (5′‐ACGACATCTGCATGAGGACCCTC‐3′) and Tubulin‐R (5′‐AACCATGTTGACGGCCAACTTCCTC‐3′).

### Statistical analyses

At least three repetitions with individual biological sample sets were done for each experiment. Means were subjected to Student's *t*‐test to evaluate the significance of any differences.

## Supporting information


**Fig. S1** Conserved 488 bp coding regions in *PVS1*, *PVS2*, *PVS3* and *PVS4* were chosen as the RNAi target, based on sequence similarity. (A) Schematic representation of coding regions in *PVS* genes. Solid vertical bars correspond to intron positions. Red bar indicates target region for RNAi. (B) Coding sequences in *PVS* genes were aligned using Clustal W, and the target region for *PVS*‐RNAi is depicted with red bars.

**Fig. S2** Transgenic plants and tubers developed normally.

**Fig. S3** Effect of water treatment on GUS activity in *PVS3p:GUS*‐expressed potato tubers and leaves at 2 days and 3 days post inoculation (dpi). (A) Surfaces of tuber slices were treated with water, then cut vertically. Tubers were observed for GUS staining. (B) *PVS3p:GUS*‐expressed potato leaves were treated with water. Stained leaves were observed using a stereoscopic microscope. Bars, 100 µM.

**Fig. S4** Expression of *HMGR2* and *PVS3* genes in potato (*Solanum tuberosum*) leaves of wild‐type (WT), RNAi‐17 (#17) and RNAi‐30 (#30) in response to an avirulent isolate of *Phytophthora infestans*. Total RNAs were extracted from leaves at 6 h or 12 h after inoculation (hpi) and were used for real time Reverse Transcription‐quantitative Polymerase Chain Reaction (RT‐qPCR). Letters represent each significance group, determined by Tukey's multiple range test. Data are means ± standard deviations (SDs) from at least three independent experiments.

**Fig. S5** Reversed‐phase high performance liquid chromatography (HPLC) analysis of phytoalexins from potato (*Solanum tuberosum*) tubers of wild‐type (WT) and RNAi‐30. Phytoalexins were extracted from tubers 1 day after inoculation with an avirulent isolate of *Phytophthora infestans*. Solvents: A, distilled water; B, acetonitrile. The dotted line indicates the gradient programme. Peaks of rishitin (*) and lubimin (**) were observed in the ultraviolet (UV) spectrum (210 nM) of WT extract at retention time of 31 min and 43 min, respectively.

**Fig. S6** Extracts from *PVS*‐silenced potato (*Solanum tuberosum*) tubers did not inhibit mycelial growth of *Phytophthora infestans*. Extracts were prepared from tubers 1 day after inoculation with an avirulent isolate of *P. infestans*. A filter paper was spotted with 20 µg of extracts and placed on rye agar media to analyse mycelial growth of *P. infestans*. The photograph was taken 5 days after co incubation with a mycelial mat of *P. infestans*. The yellow arrow indicates a clear zone showing inhibitory activity against mycelial growth.

**Fig. S7** Thin layer chromatography (TLC) and high performance liquid chromatography (HPLC) analysis of extracts from potato (*Solanum tuberosum*) leaves of wild‐type (WT) and RNAi‐30. (A) wild‐type and RNAi‐30 leaves were inoculated with an avirulent isolate of *Phytophthora infestans*. Extracts were prepared from leaves 1 day after inoculation. The extracts and 20 µg purified lubimin were separated and developed on a TLC plate. (B) These leaf extracts were further analysed by Reversed‐phase HPLC. Solvents: A, distilled water; B, acetonitrile. The dotted line indicates the gradient programme.

**Fig. S8** Extracts from potato (*Solanum tuberosum*) leaves do not inhibit mycelial growth of *Phytophthora infestans*. Extracts were prepared from leaves 1 day after inoculation with an avirulent isolate of *P. infestans*. The filter paper spotted with 50 µg of extracts was placed on rye agar media to analyse mycelial growth of *P. infestans*. The photograph was taken 5 days after co‐incubation with a mycelial mat of *P. infestans*.

**Table S1** Primer sequences for real time Reverse Transcription‐quantitative Polymerase Chain Reaction (RT‐qPCR).
