# Supplementary figures and images for: RNAi of the sesquiterpene cyclase gene for phytoalexin production impairs pre‐ and post‐invasive resistance to potato blight pathogens
Source: Mol Plant Pathol. 2019 Apr 16;20(7):907–22. doi: 10.1111/mpp.12802 (PMC6589726; doi:10.1111/mpp.12802)

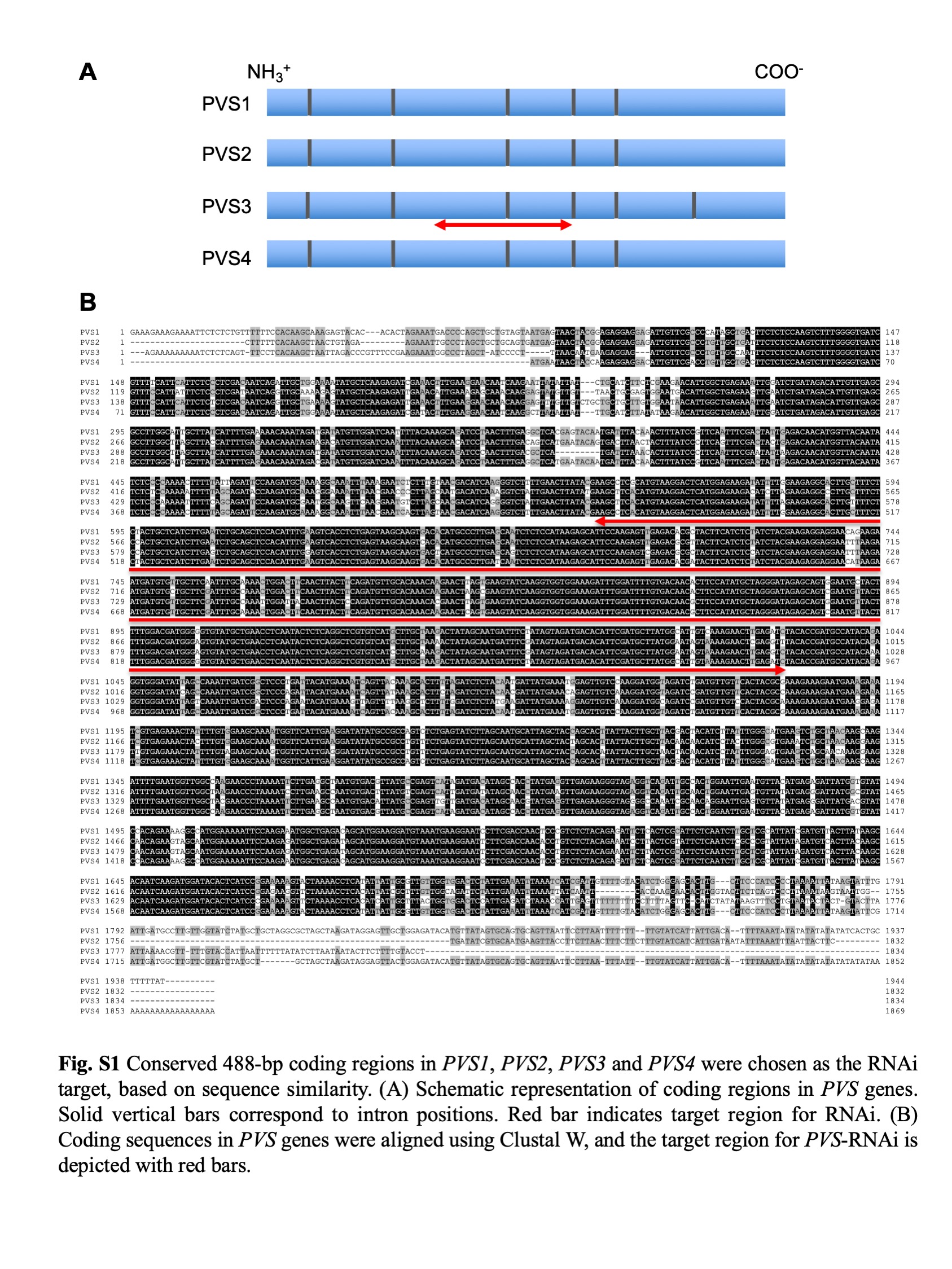

Supplement: Supplementary file 1 — Fig. S1 Conserved 488 bp coding regions in PVS1, PVS2, PVS3 and PVS4 were chosen as the RNAi target, based on sequence similarity. (A) Schematic representation of coding regions in PVS genes. Solid vertical bars correspond to intron positions. Red bar indicates target region for RNAi. (B) Coding sequences in PVS genes were aligned using Clustal W, and the target region for PVS‐RNAi is depicted with red bars. [file MPP-20-907-s001.jpg]

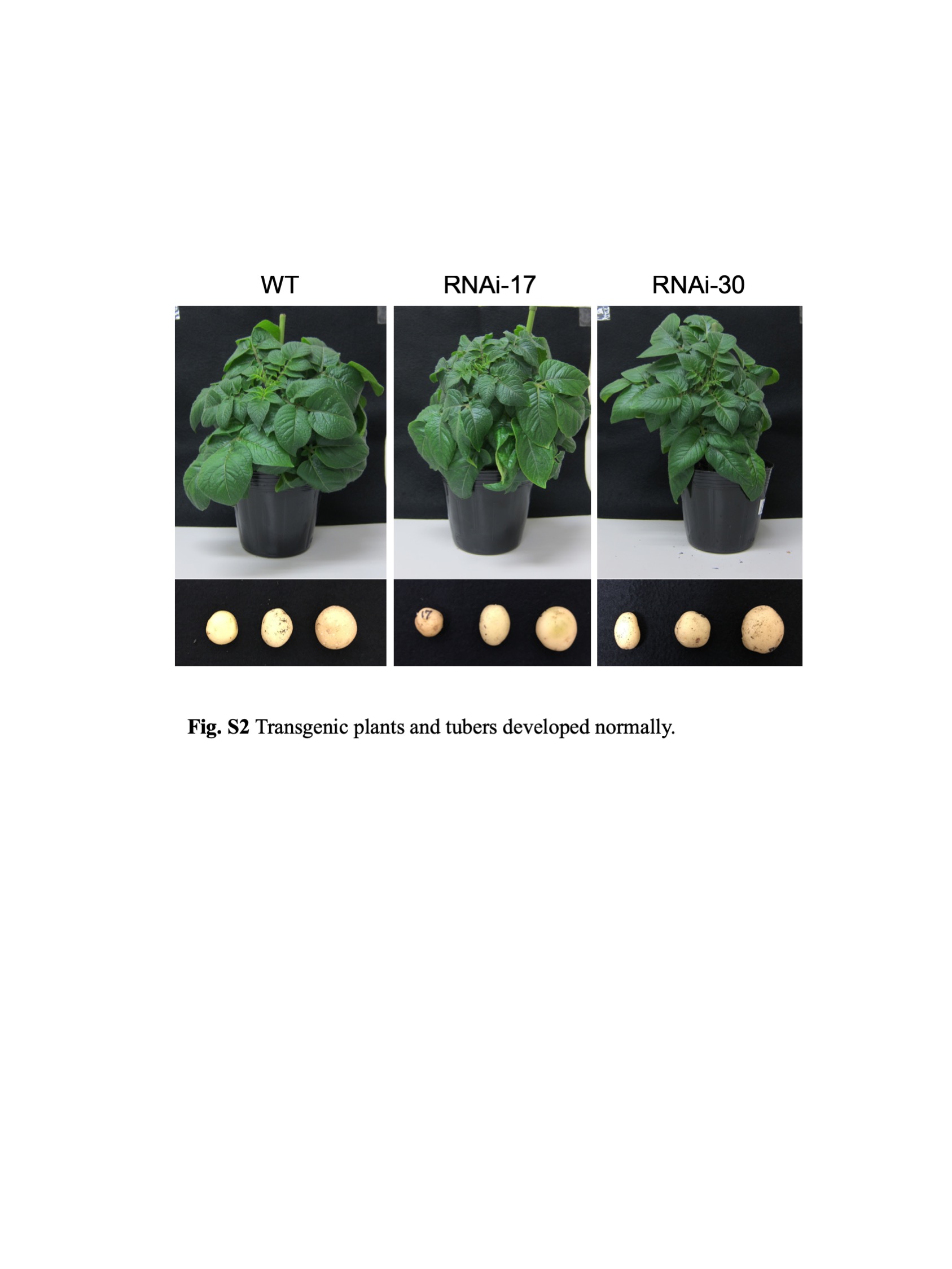

Supplement: Supplementary file 2 — Fig. S2 Transgenic plants and tubers developed normally. [file MPP-20-907-s002.jpg]

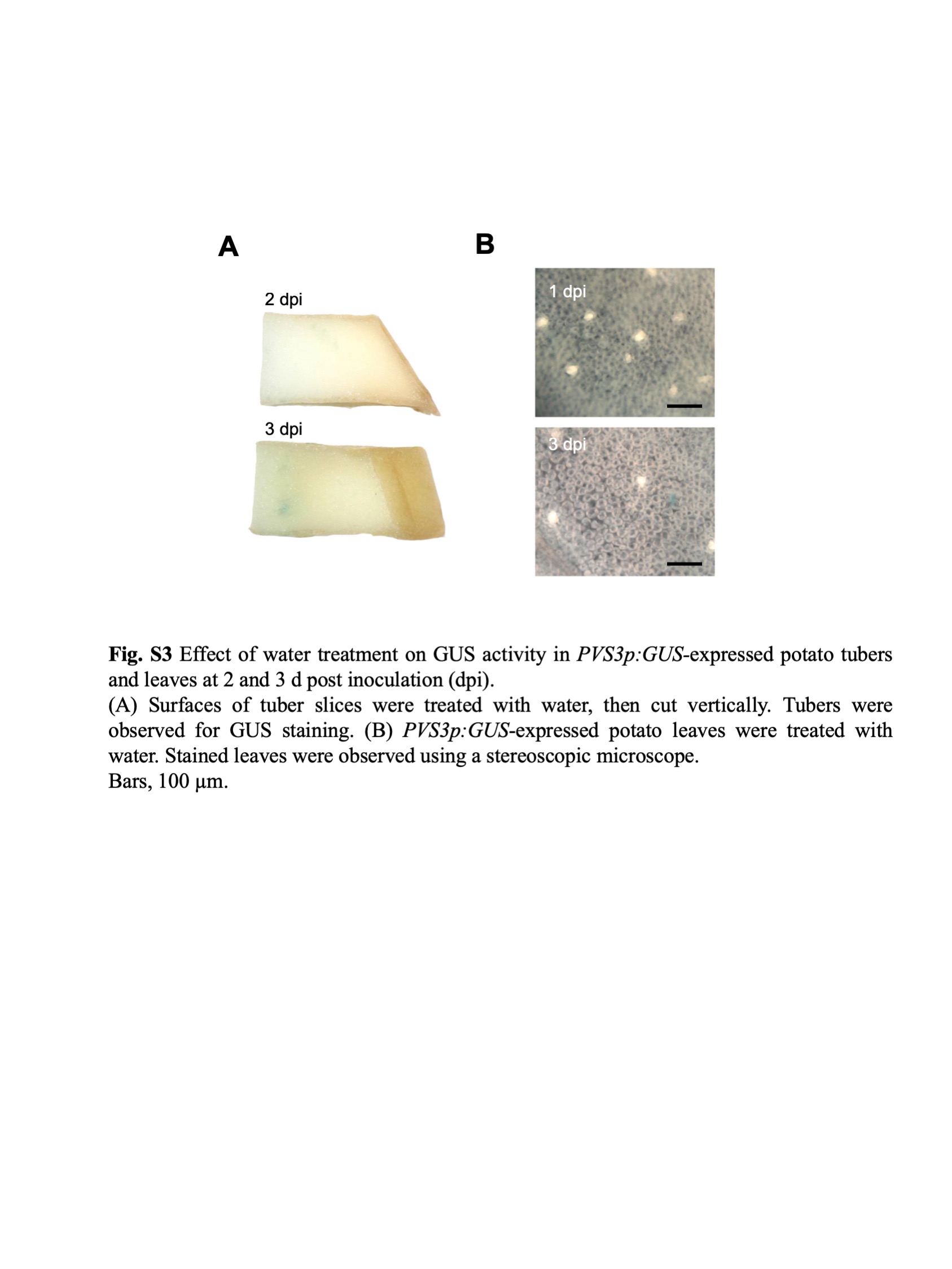

Supplement: Supplementary file 3 — Fig. S3 Effect of water treatment on GUS activity in PVS3p:GUS‐expressed potato tubers and leaves at 2 days and 3 days post inoculation (dpi). (A) Surfaces of tuber slices were treated with water, then cut vertically. Tubers were observed for GUS staining. (B) PVS3p:GUS‐expressed potato leaves were treated with water. Stained leaves were observed using a stereoscopic microscope. Bars, 100 µM. [file MPP-20-907-s003.jpg]

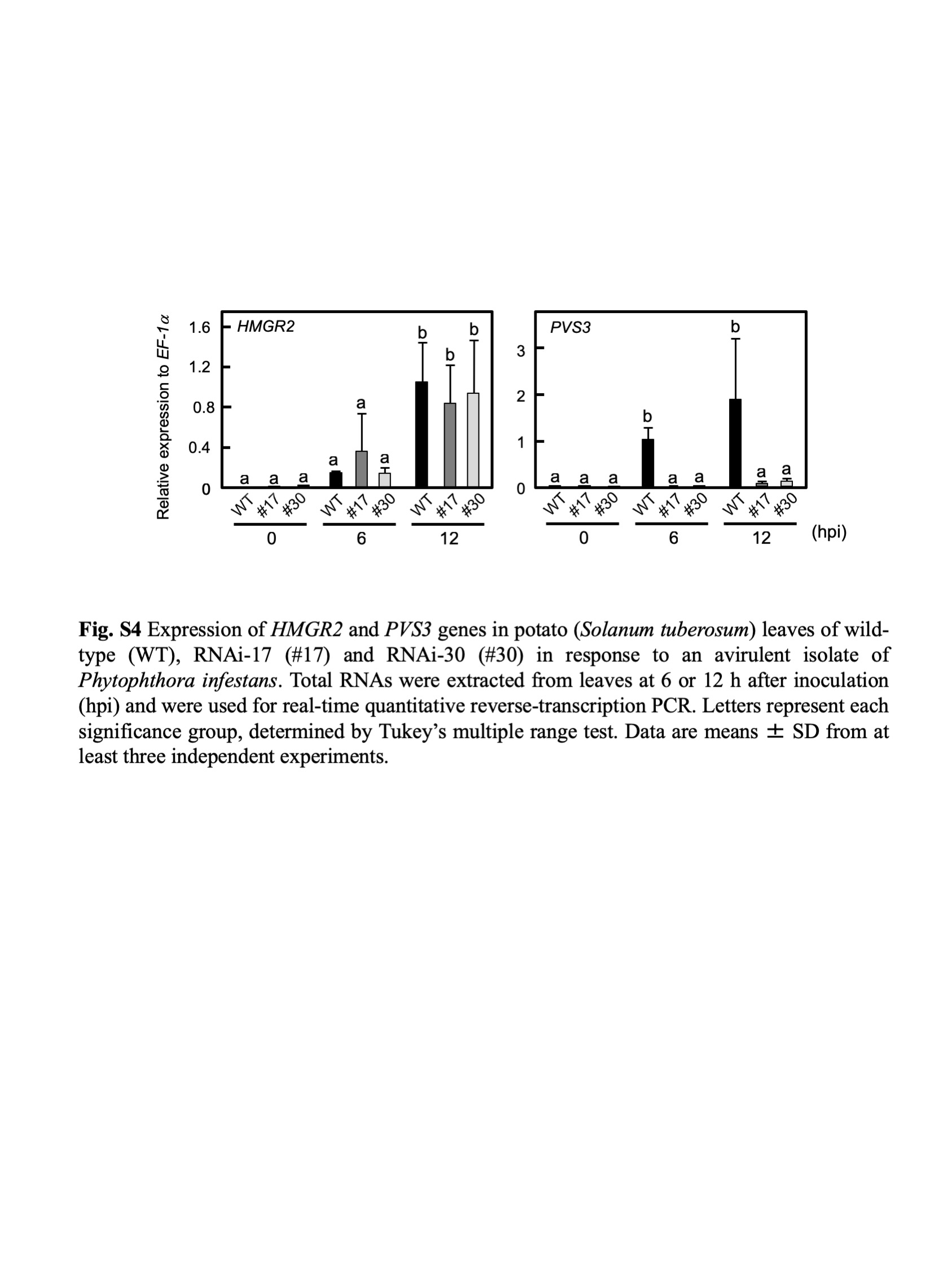

Supplement: Supplementary file 4 — Fig. S4 Expression of HMGR2 and PVS3 genes in potato (Solanum tuberosum) leaves of wild‐type (WT), RNAi‐17 (#17) and RNAi‐30 (#30) in response to an avirulent isolate of Phytophthora infestans. Total RNAs were extracted from leaves at 6 h or 12 h after inoculation (hpi) and were used for real time Reverse Transcription‐quantitative Polymerase Chain Reaction (RT‐qPCR). Letters represent each significance group, determined by Tukey's multiple range test. Data are means ± standard deviations (SDs) from at least three independent experiments. [file MPP-20-907-s004.jpg]

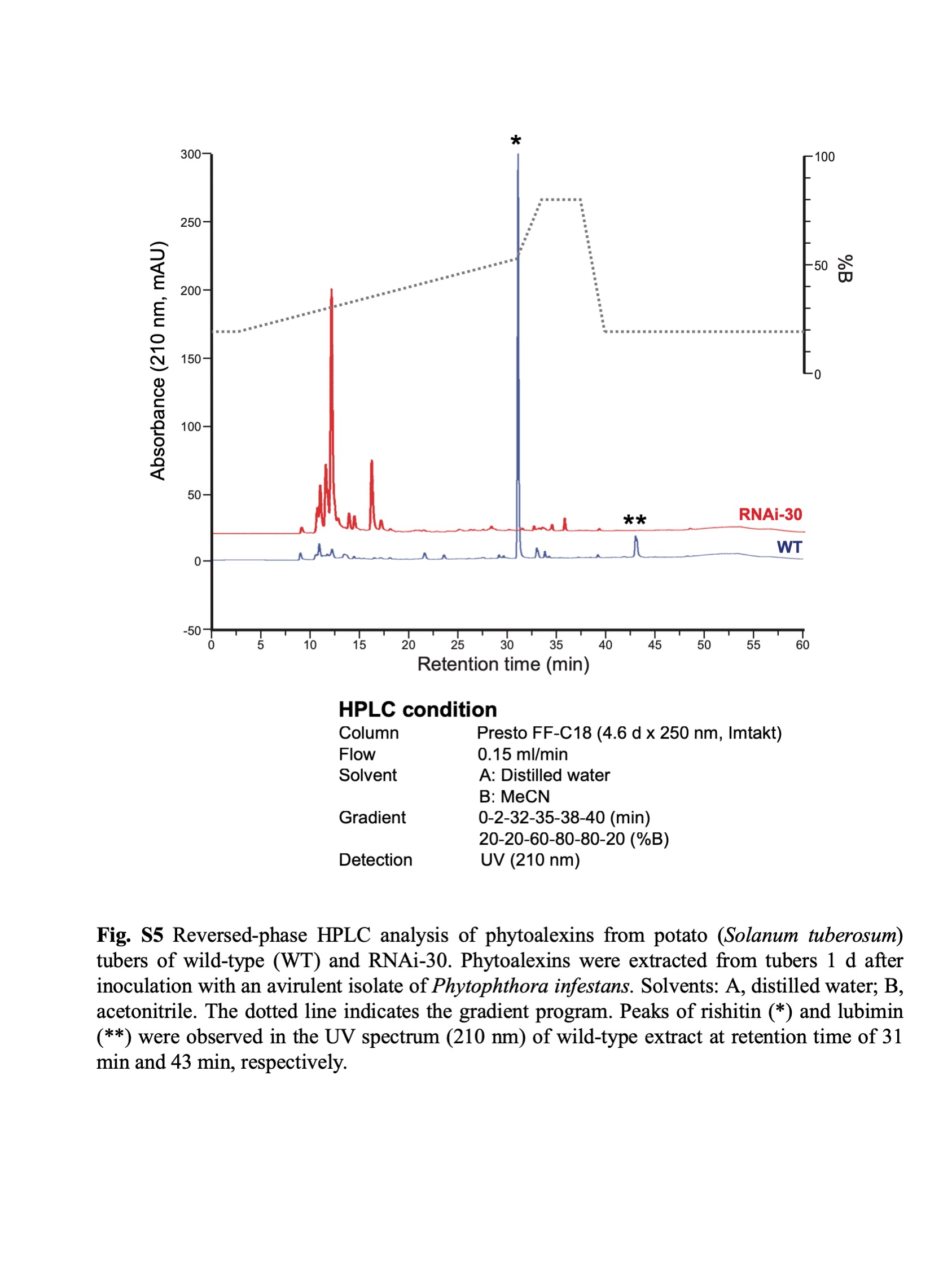

Supplement: Supplementary file 5 — Fig. S5 Reversed‐phase high performance liquid chromatography (HPLC) analysis of phytoalexins from potato (Solanum tuberosum) tubers of wild‐type (WT) and RNAi‐30. Phytoalexins were extracted from tubers 1 day after inoculation with an avirulent isolate of Phytophthora infestans. Solvents: A, distilled water; B, acetonitrile. The dotted line indicates the gradient programme. Peaks of rishitin (*) and lubimin (**) were observed in the ultraviolet (UV) spectrum (210 nM) of WT extract at retention time of 31 min and 43 min, respectively. [file MPP-20-907-s005.jpg]

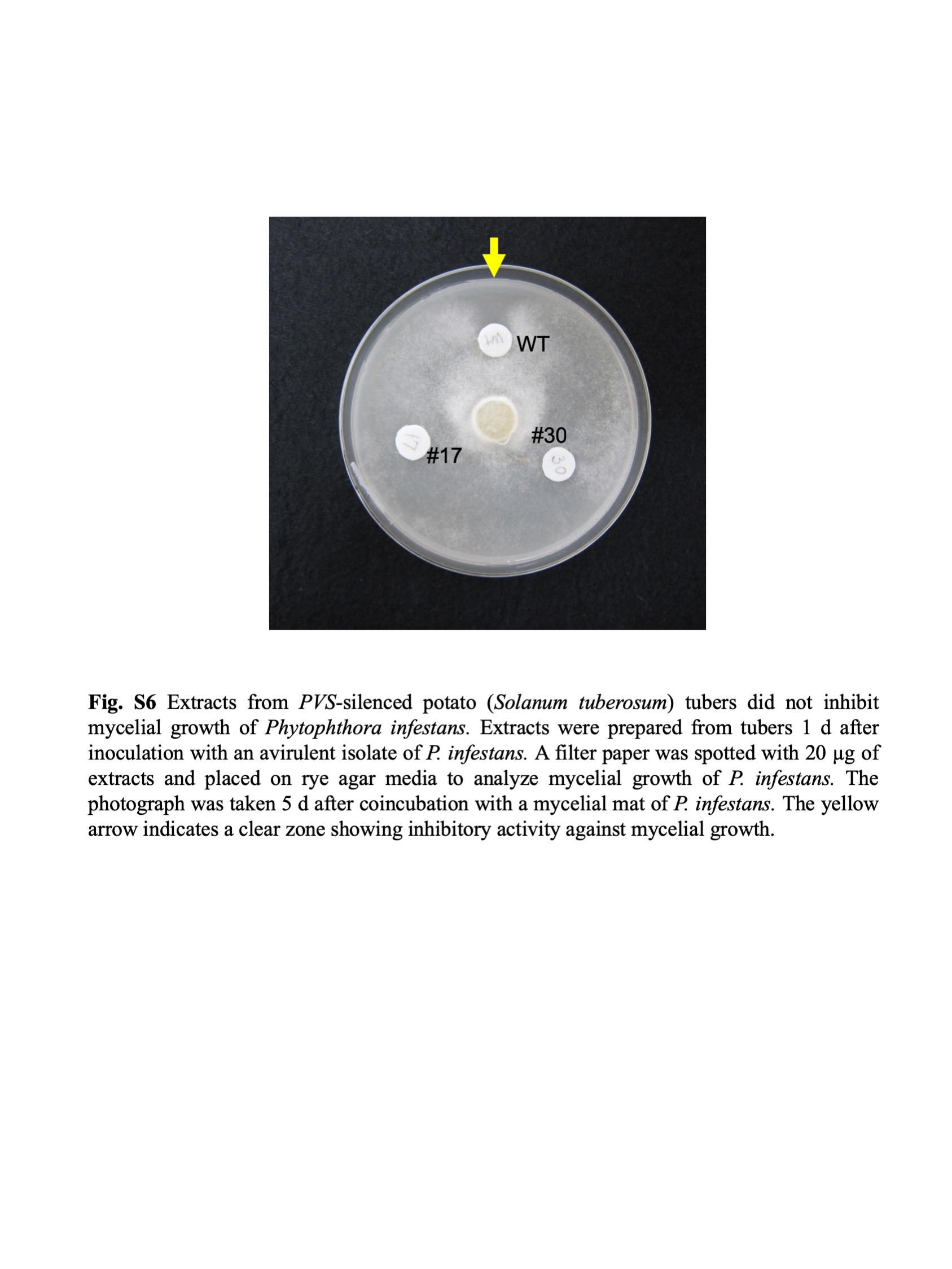

Supplement: Supplementary file 6 — Fig. S6 Extracts from PVS‐silenced potato (Solanum tuberosum) tubers did not inhibit mycelial growth of Phytophthora infestans. Extracts were prepared from tubers 1 day after inoculation with an avirulent isolate of P. infestans. A filter paper was spotted with 20 µg of extracts and placed on rye agar media to analyse mycelial growth of P. infestans. The photograph was taken 5 days after co incubation with a mycelial mat of P. infestans. The yellow arrow indicates a clear zone showing inhibitory activity against mycelial growth. [file MPP-20-907-s006.jpg]

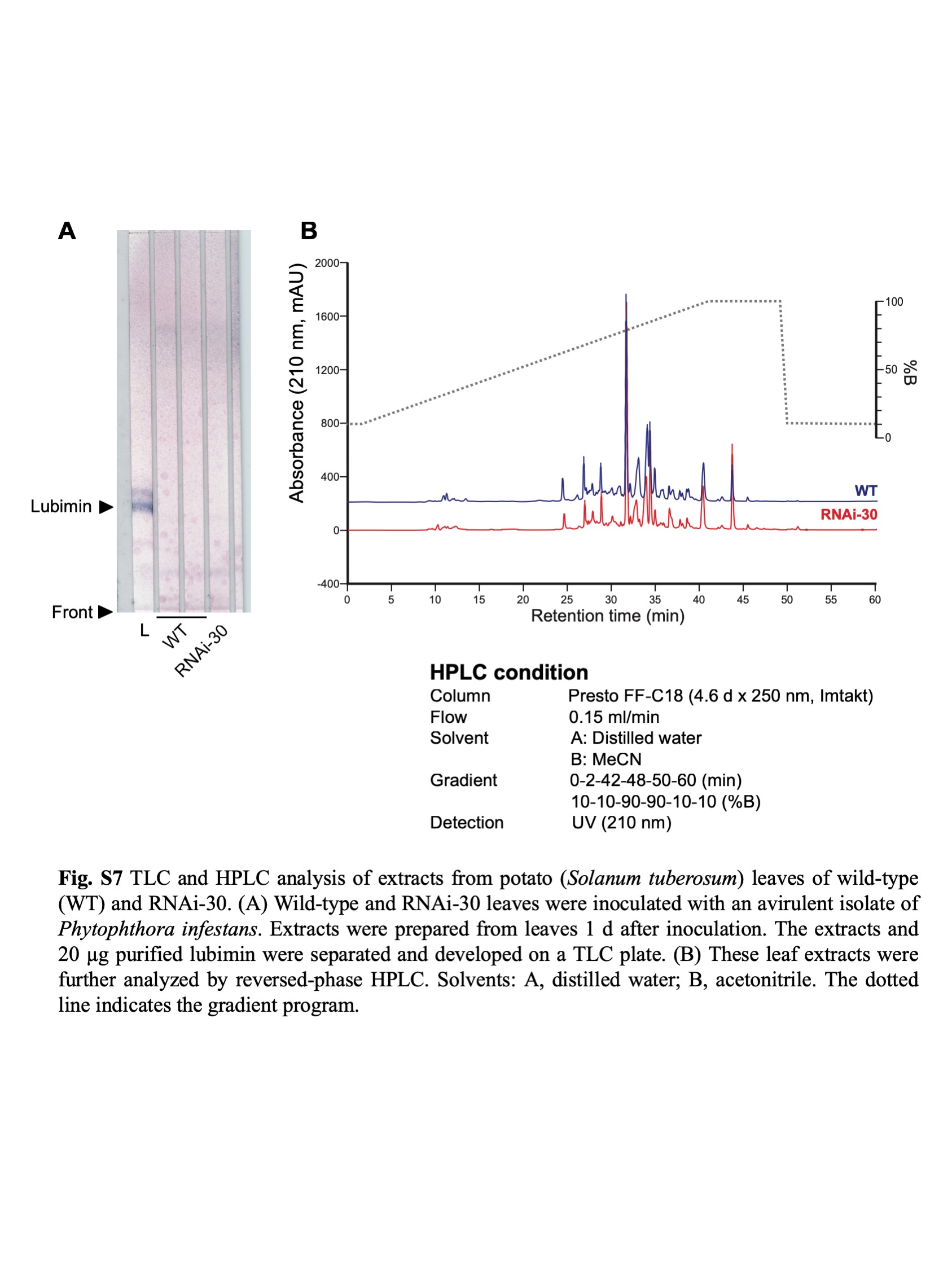

Supplement: Supplementary file 7 — Fig. S7 Thin layer chromatography (TLC) and high performance liquid chromatography (HPLC) analysis of extracts from potato (Solanum tuberosum) leaves of wild‐type (WT) and RNAi‐30. (A) wild‐type and RNAi‐30 leaves were inoculated with an avirulent isolate of Phytophthora infestans. Extracts were prepared from leaves 1 day after inoculation. The extracts and 20 µg purified lubimin were separated and developed on a TLC plate. (B) These leaf extracts were further analysed by Reversed‐phase HPLC. Solvents: A, distilled water; B, acetonitrile. The dotted line indicates the gradient programme. [file MPP-20-907-s007.jpg]

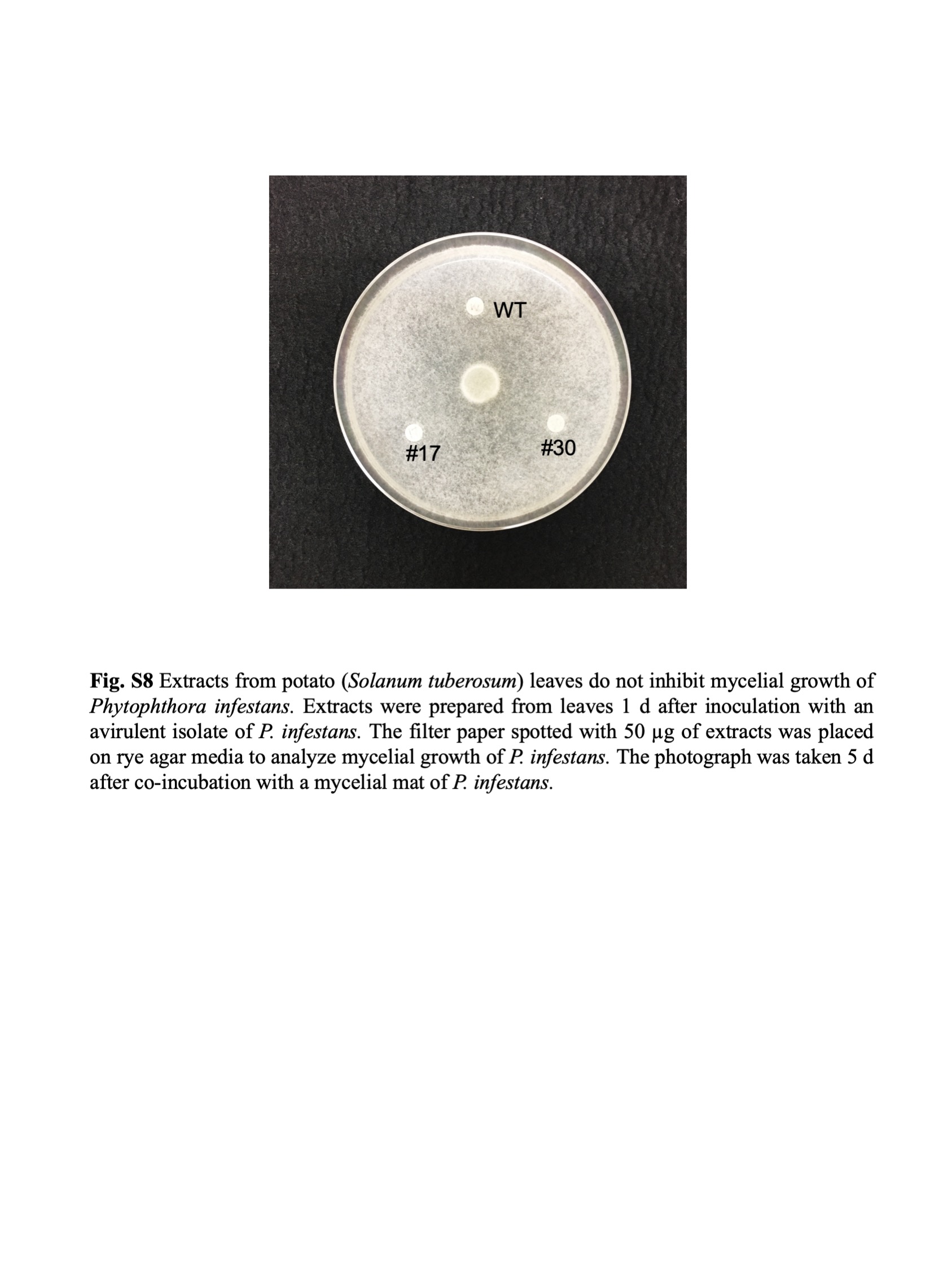

Supplement: Supplementary file 8 — Fig. S8 Extracts from potato (Solanum tuberosum) leaves do not inhibit mycelial growth of Phytophthora infestans. Extracts were prepared from leaves 1 day after inoculation with an avirulent isolate of P. infestans. The filter paper spotted with 50 µg of extracts was placed on rye agar media to analyse mycelial growth of P. infestans. The photograph was taken 5 days after co‐incubation with a mycelial mat of P. infestans. [file MPP-20-907-s008.jpg]

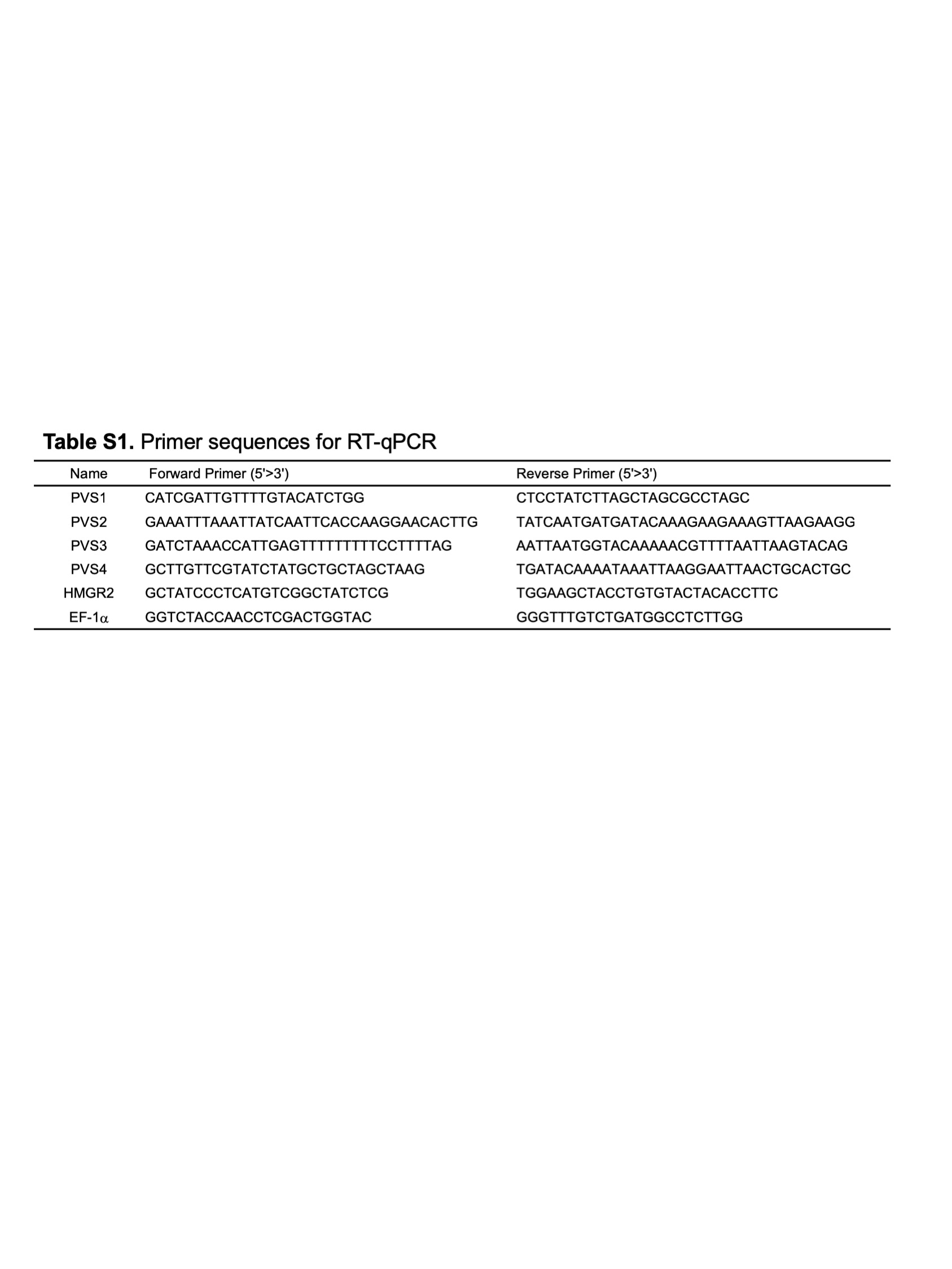

Supplement: Supplementary file 9 — Table S1 Primer sequences for real time Reverse Transcription‐quantitative Polymerase Chain Reaction (RT‐qPCR). [file MPP-20-907-s009.jpg]
